# Recommendations for developing accessible patient information leaflets for clinical trials to address English language literacy as a barrier to research participation

**DOI:** 10.1186/s13063-024-08471-5

**Published:** 2024-09-27

**Authors:** Vikki Wylde, Sharon Brennan, Emma Johnson, Kirsty Roberts, Andrew D. Beswick, Catherine Jameson

**Affiliations:** 1 Musculoskeletal Research Unit, Bristol Medical School, University of Bristol, Bristol, UK; 2 NIHR Bristol Biomedical Research Centre, University Hospitals Bristol and Weston NHS Foundation Trust and University of Bristol, Bristol, UK; 3 National Voices, London, UK; 4 Bristol Trials Centre, Bristol Medical School, University of Bristol, Bristol, UK

**Keywords:** Patient information leaflet, Clinical trials, Recommendations, Accessible, Easy-read

## Abstract

**Background:**

Low English language literacy is a common barrier to participation in clinical trials. Patient information leaflets (PILs) used in clinical trials are often lengthy, complex and have poor readability; this is a persistent and prevalent problem common to trials across the world. Simplifying the information provided in PILs can lead to improved understanding, comprehension and knowledge.

The aim of this project was to develop recommendations for developing accessible PILs for clinical trials through a literature review of published and grey literature and co-working with marginalised communities, patients, and health and social care charities.

**Methods:**

A literature review of MEDLINE, Embase and online resources was conducted, and recommendations for developing accessible PILs were extracted from eligible published and grey literature. Grey literature which contained insights into more inclusive forms of communication was also identified and summarised. Meetings were held with two racially marginalised community groups, two groups involving autistic adults and/or adults with learning difficulties and a patient advisory group. Examples of accessible PILs were shared and discussions held about the content and format of the PILs and suggestions for changes/improvements. National Voices, a coalition of health and social care charities in England, held a national online workshop with charities and lived experience partners. Recommendations identified from the multiple sources were coded, collated and refined to develop an overarching framework of recommendations.

**Results:**

The framework consists of 74 recommendations for developing accessible PILs for clinical trials. Recommendations cover the five topics of formatting, information presentation, writing style, content and accessibility.

**Conclusions:**

This project has developed a comprehensive framework of recommendations to guide researchers in the development of accessible PILs for clinical trials. Findings from previous research and from co-working with marginalised communities, patients and health and social care charities were collated to ensure that a diverse range of voices and experiences informed the framework. These recommendations aim to support researchers to develop better study information to reduce English language literacy as a barrier to participation in clinical trials.

**Supplementary Information:**

The online version contains supplementary material available at 10.1186/s13063-024-08471-5.

## Introduction

It is now widely acknowledged that action needs to be taken to improve diversity and inclusion in clinical trials and health research more broadly [1]. Trial sample populations need to reflect the communities that they serve to ensure equity, scientific integrity, a full understanding of differences in treatment responses, safety of new treatments, and the translation and applicability of findings into real-world application [2]. The imperative for more inclusive practices in clinical trials was highlighted during the COVID-19 pandemic, with a widespread lack of diversity in people participating in vaccine trials despite Black and Asian ethnic groups having a higher risk of death from COVID-19 [3]. There are a number of national- and government-level initiatives focussed on addressing the underrepresentation of diverse populations in clinical trials, such as the UK National Institute for Health Research (NIHR) Innovations in Clinical Trial Design and Delivery for the underserved (INCLUDE) project [4], Trial Forge [5] and the USA Clinical Trials Transformation Initiative [6]. However, the underrepresentation of marginalised groups in health research prevails due to multi-faceted barriers to research participation. The barriers experienced vary across marginalised groups and individuals but have broadly been identified as relating to language and communication, lack of trust, eligibility criteria, attitudes and beliefs, lack of knowledge around clinical trials and logistical and practical issues [7]. Specific to language and communication, low English language literacy levels are a well-known barrier to inclusion in clinical trials [7], relevant to different marginalised groups including people with a lower education level, those who do not read written English, have a learning disability, are living with dementia or who have had a stroke.

The National Literacy Trust estimates that 7.1 million people (16% of adults) living in England have very poor literacy [8]. Numerous studies have found that patient information leaflets (PILs) used in clinical research are often lengthy, inappropriately complex and have poor readability; this is a persistent and prevalent problem common to trials across the world [9–12]. For example, an evaluation of COVID-19 vaccine trials found the mean word count of PILs was 8333 words (average reading time of 35–48 min) and the language complexity was high [13]. There are substantial concerns about the increasing length and complexity of PILs for clinical trials and the potential impact on people’s comprehension of the information provided [14]. This also can pose challenges to translation of study information into different languages. Simplifying the information provided in PILs can lead to improved understanding, comprehension and knowledge [15–17]. ‘Easy read’ has been defined by as information which is written using simple words supported by images [18]. Information presented in an ‘Easy read’ format aims to be easier to understand than standard documents and can be beneficial for a range of audiences.

The aim of the MAPLE (*M*aking trials more *A*ccessible through better *P*atient information *LE*aflets) project was to develop recommendations for developing accessible PILs for clinical trials through a literature review of published and grey literature and co-working with marginalised communities, patients and health and social care charities.

## Methods

The UK standards for Public Involvement in research defines it as ‘research being carried out ‘with’ or ‘by’ members of the public rather than ‘to,’ ‘about’ or ‘for’ them’ [19]. The UK National Institute for Health Research provides ‘commenting on and developing patient information leaflets or other research materials’ as an example of patient and public involvement in research [20]. This project involved working with members of the public to develop recommendations for developing accessible PILs for clinical trials, and therefore was conducted as public and community involvement and engagement (PCIE) activities, rather than research, and institutional ethics approval was not required.

This project was a partnership between academics at the University of Bristol and National Voices. National Voices (https://www.nationalvoices.org.uk/) is a leading coalition of health and social care charities in England. They have more than 200 members covering a diverse range of health conditions and communities, connecting them with the experiences of hundreds of thousands of people.

### Literature review of published and grey literature

As this was a literature review rather than a systematic review, the review protocol was not registered on PROSPERO.

#### Published literature

In designing a search strategy, we acknowledged that searching for studies relating to ‘patient information’ would be highly unspecific and identify a large quantity of irrelevant material and searches for ‘patient information leaflet’ would identify some relevant literature but may miss material addressing the issue with a broader consideration of the delivery of patient information. To address this, we applied both a search of online databases with a strategy based around patient information leaflets and a snowballing method with forward searching based on citations of key studies [21]. For a search of MEDLINE and Embase on the Ovid platform on 16th November 2023, we used a search based on textwords used in the review of Sustersic and colleagues [22] and a filter for randomised controlled trials and controlled clinical studies (see Supplementary Table 1). Risk of bias of included studies was not assessed.

To identify articles citing key publications, we used the citation tracking option in Web of Science. Initially, we focused on six key publications that we were aware of [22–27], and after screening of reference lists and forward citations, we tracked 22 studies [9, 11, 17, 22–40]. Articles were included if they reported recommendations to inform the development of easy-read clinical trial PILs for adults. No limitations were placed on the study design. The scope of included articles was limited to recommendations focused on research; studies related to the development of PILs for clinical care were excluded. Article titles were screened in Endnote and clearly irrelevant articles were excluded. Abstracts and full text of potentially relevant articles were then screened to determine eligibility. Screening was performed by one reviewer.

Data extraction of recommendations from included articles was performed by one reviewer and comprised author, date, study design and recommendation. Recommendations were extracted verbatim, and extracted data were entered in Excel.

#### Grey literature

In November 2023, a search of grey literature of potential relevance was conducted through searches of online material published or catalogued by the King’s Fund, Care Quality Commission, Healthcare Quality Improvement Partnership and Health Research Authority. Opengrey and Google were also searched. Grey literature identified from eligible articles was also included.

To supplement the search of the grey literature, National Voices utilised knowledge and networks of equalities-focussed charities to identify grey literature which contained insights into more inclusive forms of communication. This included reflections on the innovations that could be used to ensure people with specific communication needs have an equal opportunity to participate in clinical research, including people with sensory impairments, those with learning disabilities, autistic people, those living with dementia, and people with low or no literacy or those who do not speak English fluently. This included literature specific to clinical trial participation as well as innovative work on how to improve and create accessible communications regardless of the subject matter.

### Co-working with marginalised communities, patients and health and social care charities

#### Marginalised communities and patient groups

Following our co-produced guidance on inclusive involvement of community groups in health research [41], we co-worked with two racially marginalised community groups, two groups involving autistic adults and/or adults with learning disabilities and/or difficulties and one patient advisory group to generate recommendations for designing accessible PILs. An overview of the groups and meetings is provided in Table 1. Each meeting lasted 1–4 h and was held online or in the usual venue of the group and followed each group’s preferred format, with English interpretation provided for the researchers by the community leaders/facilitators as needed. Meetings were facilitated by group leaders, with researchers in attendance. Groups were reimbursed for their involvement by their preferred format [41]. All meetings were held for the purposes of this project, with the exception of the four meetings with The Adventurers. Three of these meetings focussed on co-developing an accessible PIL for a clinical trial and the fourth meeting involved a discussion about supporting research participation; with permission, notes and learning from those meetings were used in this project.

**Table 1:** Overview of community groups and meetings

Group name	Group description	Number of meetings	Number of attendees at meeting(s)	Format
Dhek Bhal	Charitable organisation that provides support and care for the older South Asian community in Bristol	2 (one meeting with men’s group, one meeting with women’s group)	15–20 members for women’s group, 5–10 members for men’s group	In person
My Friday coffee morning	A group for women resident in the Barton Hill area of Bristol to meet and discuss relevant social and health issues, with membership being majority women of Black, African and Caribbean heritages	2	12 (over the two meetings)	In person
Lawnmowers Independent Theatre Company – research abilities group	Research Abilities, set up in partnership with the Lawnmowers Independent Theatre Company, is a Public and Patient Involvement and Engagement group based in the North East of England, comprising members with learning difficulties	1	7 members and 1 staff member	Online
The Adventurers	A panel of experts by experience comprising autistic people and people with learning disabilities from across the South of England, supported by the charity Brandon Trust	4	4–7 members and 2–3 staff members/support workers per meeting	In person and online
Patient Experience Partnership in Research (PEP-R)	Patient and public involvement group at the University of Bristol comprising mostly older adults living with long-term musculoskeletal conditions	1	6 members and 1 facilitator	In person

To inform the discussion during the meetings, a selection of example accessible PILs was obtained through the Bristol Trials Centre, and consent was gained from the trial teams to share the accessible PILs with community and patient groups. For each meeting, 2–3 accessible PILs were printed, and copies were shared with members to facilitate discussion. The agenda for the meetings were informal and adapted to the preferences for working of each group, to allow people the time and space to contribute their experiences to open discussion. Discussions focussed on whether the PILs were easy to understand, what people liked/disliked about them, what would make them better, and whether more/less information should be included. A researcher took notes of the discussion during each meeting, rather than audio-recording, to ensure that the group members felt comfortable to openly share their thoughts and views with the researchers.

#### Health and social care charities and lived experience partners

National Voices convened and facilitated a 1-h online workshop with 18 people, comprising a mixture of professionals working in health and social care charities and people with lived experience of long-term health conditions and/or disability. Health charities represented during the workshop were The Nerve of My Multiple Sclerosis, Macular Society, TransActual, Thomas Pocklington Trust, Roma Support Group, South Asian Health Action, BHA For Equality, Blood Cancer UK, British Heart Foundation, Age UK, and Rethink Mental Illness. A further five individuals were consulted individually in follow-up conversations. The workshop focused on reviewing barriers to participation and, asking participants to identify the key information researchers would need to include in an accessible format, and identifying solutions and approaches to ensure the proposed output meets the needs of people who are underrepresented in current research. A full report of the workshop is available on the National Voices website [42].

### Analysis

Development of the framework of recommendations for the creation of accessible PILs was an iterative process. Extracted recommendations from articles and documents identified in the literature review were coded in Excel and grouped into topics by one researcher (VW). These preliminary codes were then reviewed by a second researcher (AB). Notes from the community and patient group meetings were then reviewed line-by-line by one researcher (VW) and coded in Excel, using the provisional framework developed from the literature review data. New codes were added as they arose, and existing codes refined during the coding process. This process was repeated for the National Voices report on inclusive communication and the report from the online workshop with health and social care charities and lived experience partners. The amalgamated matrix of coded recommendations, along with the supporting section of notes from the meetings, was then reviewed and refined by a second researcher (CJ). The overarching framework was then reviewed by all the co-authors to merge duplicate categories, review the topic categories and finalise the order of presentation of the recommendations.

## Results

**Fig. 1:**
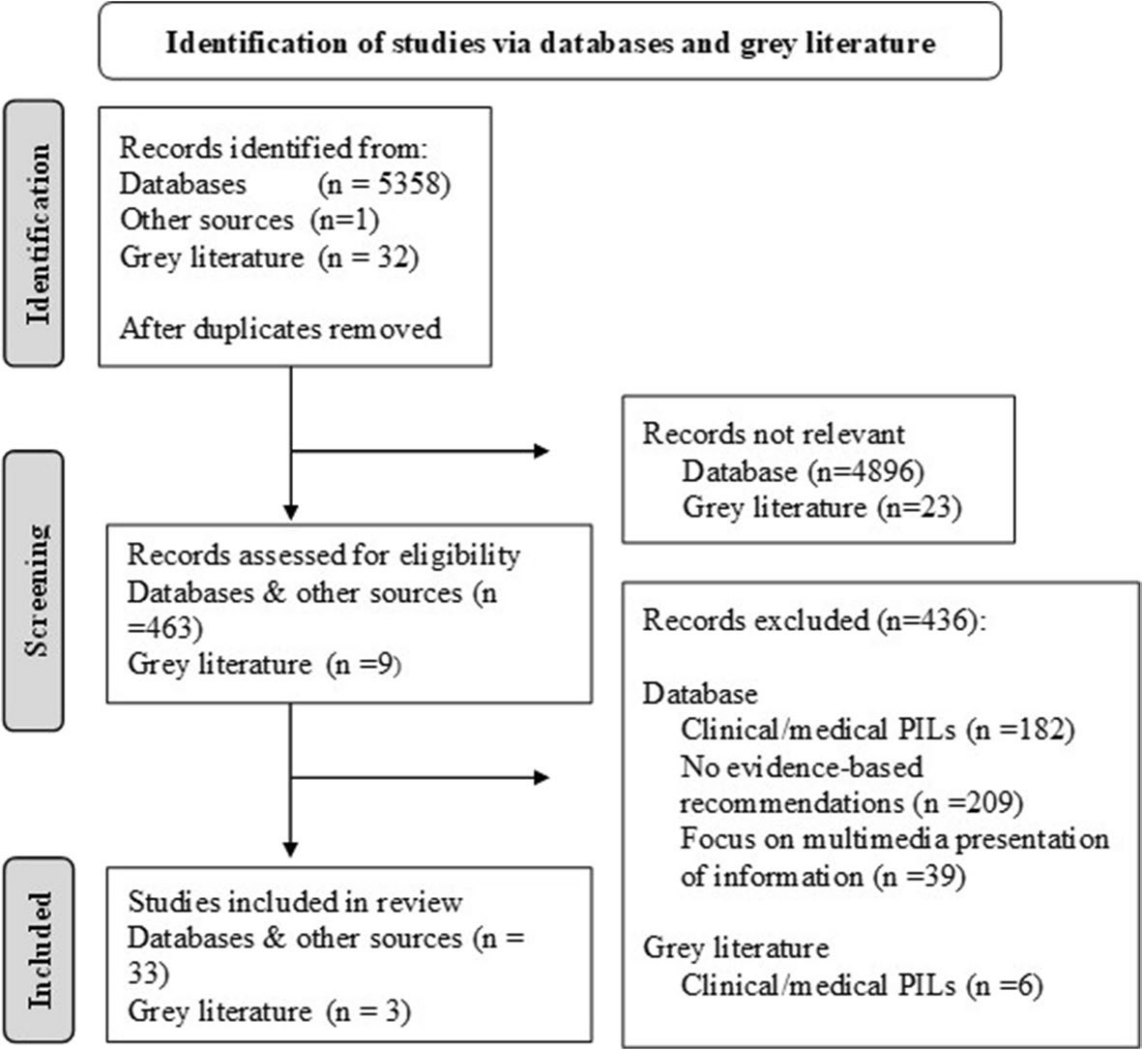
Literature review flow diagram

A flow diagram of the literature review is provided in Fig. 1. Database searches identified 5358 articles after duplicates were removed and another article was identified through direct discussion with the trial team. After initial screening, 4896 articles were removed as they were irrelevant and 462 were screened in-depth; of these 33 were included [7, 11, 15, 24, 28, 32, 34, 36, 43–67]. A summary of the characteristics of the included studies is provided in Supplementary Table 2 and the extracted recommendations from studies are presented in Supplementary Table 3. The grey literature search identified 32 online documents of which 9 were screened in depth and 3 were included [68–70]. Recommendations identified from the literature review of published and grey literature, review of grey literature by National Voices, community and patient group discussions, and the workshop with health and social care charities and lived experience partners were collated and brought together in an overarching framework of recommendations for developing accessible clinical trial PILs. This framework consists of 74 recommendations, grouped into five overarching topics of formatting, information presentation, writing style, content and accessibility. These are further divided into 31 subtopics to facilitate navigation of the framework. The recommendations are provided in full in Table 2 and summarised below.

**Table 2:** Recommendations for developing accessible PILs for clinical trials

Topic	Recommendations	Source of recommendation
**Formatting**		
Alignment	Use left-aligned text	Literature review
Break up text	Avoid blocks of text; break the text up into bullet points, lists, short sections and/or present information in boxes or use text boxes to highlight key information	The Adventurers, literature review
Colour	Use colour, with consideration for contrast and readability e.g. use dark letters on a light background	The Adventurers, literature review
Columns	Use two columns, with images on left-hand side and text on right-hand side	The Adventurers, My Friday Coffee Morning, literature review, National Voices workshop
	Use columns with a line length of 40–50 characters to ensure enough separation between columns to sufficiently separate the text	literature review
Headings	Use headings that are easily distinguished from the body of the text e.g. use bold typeface, larger font size, different colour, or place header in coloured box	The Adventurers, literature review, Lawnmowers
	Keep headings short	Literature review
	Use questions in section headings	Literature review
	Place headers as close as possible to the text	Literature review
	Use consistent formatting for each header of the same level	Literature review
	Leave more space above headings and subheadings than below them	Literature review
Paper	Make it clear if people need to turn overleaf	My Friday Coffee Morning
	Use appropriate page size, e.g. A4 paper	The Adventurers
	Use booklet format	Literature review
	Use low-to-no gloss paper	Literature review
Typeface	Use a large font size (minimum font size of 12–14) that can be modified, e.g. increased for people with visual impairment	The Adventurers, My Friday Coffee Morning, literature review, Lawnmowers
	Use a black sans serif font (e.g. Arial, Verdana or Tahoma) or fonts that have wider typefaces (e.g. Helvetica and Open Sans or Lucida Sands)	The Adventurers, literature review
	Avoid underlining, all capitals and italics and only use bold in the main text for emphasis or to highlight the main message	The Adventurers, literature review
Whitespace (space without text)	Include white space (10–35%), e.g. use wide margins and sufficient space between lines of text (1.2–1.5)	The Adventurers, literature review
**Information presentation**		
Information structure	Use a layered/tiered approach to provide information	Literature review, National Voices workshop
	Include the key messages/important information first	Literature review
	Ensure the order of the information is logical	Literature review
	Group related information together and focus on one message at a time: limit to one idea per paragraph	Literature review
	Include summaries, e.g. an ‘at a glance’ guide to study visits/data collection points, a single overall summary on the first or last page or a summary at the end of each section	Literature review
Information volume	Keep short; limit the number of messages and amount of information presented and only include information that the reader would want or need to know; avoid unnecessary information and repetition	Dhek Bhal women’s group, Dhek Bhal men’s group, PEP-R, The Adventurers, My Friday Coffee Morning, literature review, National Voices workshop, Lawnmowers
	Include enough information for people to make an informed decision	Dhek Bhal men’s group, Dhek Bhal women’s group, My Friday Coffee Morning, National Voices workshop
Images	Use images that are relevant to the trial, explain the text, support the main messages of the PIL, and/or explain a difficult concept. Consider the visual literacy of the target audience as images may make information harder to read	My Friday Coffee Morning, Dhek Bhal women’s group, PEP-R, The Adventurers, literature review, National Voices workshop, Lawnmowers
	Use images that will be recognised by the audience and consider the most appropriate type of image to use, e.g. cartoons/graphics/photographs	My Friday Coffee Morning, The Adventurers, literature review
	Use inclusive and culturally relevant images of people, ensuring that diversity is represented	Dhek Bhal women’s group, My Friday Coffee Morning, literature review, National Voices workshop
	Have one message per image and place images next to the corresponding text	The Adventurers, literature review, National Voices workshop
	Use clear, high-resolution images that are appropriately sized, explained and accompanied by a caption/label and numbered if showing a sequence	The Adventurers, literature review, Lawnmowers
	Make sure the background does not distract from the main message of the image, avoid unnecessary details in images and use cues to point out key information in an image	Literature review
Use of numbers and statistics	Limit the use of statistics; if used, check that numbers and mathematical concepts (e.g. risk, normal and range) are explained and that readers do not have to perform calculations	Literature review
	Use images and analogies to explain numbers and statistical concepts, e.g. risk	Literature review, National Voices summary
	Use whole numbers to explain risk or benefits rather than percentages	Literature review
	Using absolute risk rather than relative risk. For example, the absolute risk of an event increases from 1 in 100 to 2 in 100, but the relative risk of the event doubles	National Voices summary
	Considering using both positive and negative framing, such as ‘3 out of 100 people experienced this side effect, but 97 out of 100 did not’	National Voices summary
	Use specific amounts	Literature review
	For the numbers 0–9, use their words, for 10 + use the digit, unless you are giving an example using a statistic	Literature review
**Writing style**		
Co-production	Co-work with diverse communities to develop accessible PILs	Literature review, National Voices workshop
Inclusive language	Use inclusive phrasing and preferred terminology of target population, including everyday, familiar and culturally appropriate analogies that are clear and easy to interpret	My Friday Coffee Morning, The Adventurers, literature review, National Voices summary
Plain language	Avoid unnecessary jargon and explain terms in simple, familiar clear and precise language; avoid potentially misunderstood words/words with multiple or nuanced meanings. Minimise the use of abbreviations and acronyms and where they are necessary explain them immediately and clearly	Dhek Bhal women’s group, PEP-R, The Adventurers, literature review, National Voices summary, National Voices workshop, Lawnmowers
	Write at the appropriate reading age for the target population (generally no higher than reading age of 11–12-year-old) and assess readability through user testing and readability assessment tools	Literature review, National Voices workshop
	Avoid assumptions and patronising language	National Voices summary, National Voices workshop
	Write in a conversational and narrative style with a positive and encouraging tone demonstrating respect and value for your audience	Literature review, National Voices workshop
	Use short words, sentences and paragraphs; words of three or fewer syllables, sentences of 15–20 words or less (and avoid adding information using a subordinate clause), and paragraphs of 3–5 sentences	The Adventurers, literature review, National Voices summary
	Write from the readers’ perspective; approach the information to be provided from the point of view of what the reader wants and needs to know, rather than what the researchers think they need to convey	Literature review, My Friday Coffee Morning
	Use the active voice (aim for 80–90% active verbs)	Literature review
	Context should be provided before giving new information	Literature review
	Ensure words and terminology are consistent throughout	Literature review
	Avoid talking about 'risk' when describing participation	The Adventurers
**Content**		
Study purpose and invitation	Title the PIL ‘information leaflet’, unnecessary to include ‘easy read’	The Adventurers
	Place study logos at top of page and avoid using too many logos	The Adventurers, National Voices workshop
	Describe the purpose of clinical trials	National Voices workshop
	Describe the primary purpose of the study, providing information about the clinical condition	Dhek Bhal women’s group, literature review, National Voices workshop, Lawnmowers
	Ask if people would like to take part and emphasise that taking part is their choice; encourage discussion with other people before deciding whether to participate	Dhek Bhal women’s group, The Adventurers, PEP-R, Dhek Bhal men’s group, My Friday Coffee Morning, Lawnmowers
Importance of research and research participation	Explain the importance of participation in research; make it clear why it is valuable for that person to take part so they can understand how the research will help their community	National Voices summary, Dhek Bhal women’s group, Dhek Bhal men’s group My Friday Coffee Morning, National Voices workshop, Lawnmowers
	Explain uncertainty—communicate the uncertainty of data or if existing evidence is of low quality	National Voices summary, National Voices workshop
	Pre-empt misunderstandings—if something is easily misunderstood, or there is a common myth around a research topic, it is better to address it head-on so people feel enabled to ask questions	National Voices summary
Eligibility	Be clear about why people have been invited to take part and explain eligibility criteria	My Friday Coffee Morning, PEP-R, The Adventurers, Dhek Bhal women’s group
Randomisation	Explain treatment allocation	Dhek Bhal women’s group, The Adventurers
Treatment	Include information about research study treatment(s) and standard care and potential treatment side effects	My Friday Coffee Morning, The Adventurers, Dhek Bhal women’s group, Dhek Bhal men’s group, National Voices workshop, Lawnmowers
Study processes and data collection	Explain study processes, what will happen at each stage of the study and the time commitment	Dhek Bhal women’s group, My Friday Coffee Morning, Dhek Bhal men’s group, PEP-R, The Adventurers
	Explain processes for follow-up and data collection, including the different ways that data will be collected (e.g. phone, paper, online). If available, explain that questionnaires can be completed with help/support from the research team	My Friday Coffee Morning, Dhek Bhal women’s group, PEP-R, Dhek Bhal men’s group, The Adventurers
Data handling and confidentiality	Explain about collection and use of personal information and how confidentiality will be assured. State under what circumstances confidential data may be released and to whom and encourage questions from possible participants to surface any concerns	The Adventurers, National Voices summary, National Voices workshop
Advantages and disadvantages of participation	Explain good and bad aspects of participation	The Adventurers, My Friday Coffee Morning, Lawnmowers
Incentives	Describe any incentives or payments for participation, explaining that this it to recognise the importance and value of people’s time and effort	National Voices workshop
Withdrawal	Explain withdrawal processes	Dhek Bhal women’s group, Lawnmowers
Ethics	State the ethical standards under which the study has been devised	National Voices summary
Contact information	Provide contact information for the research team and an independent contact/advocate. Provide more than one way to contact the research team	My Friday Coffee Morning, Dhek Bhal women’s group, Dhek Bhal men’s group, PEP-R, The Adventurers, National Voices workshop, Lawnmowers
**Accessibility**		
Translation	Translate PIL into different languages and ensure interpreters and communication support is available for written and verbal information	My Friday Coffee Morning, Dhek Bhal women’s group, Dhek Bhal men’s group, National Voices summary, National Voices workshop
Alternative formats	Ask people from the beginning about their preferred means of communication of trial information and provide information in multiple formats e.g. braille, large print, plain text, audio, video format with voiceover/subtitles	The Adventurers, My Friday Coffee Morning, National Voices summary, National Voices workshop, Lawnmowers
Verbal information accompanying the PIL	Research staff need to take the time to build trust with potential participants; this may involve multiple conversations. Provide a PIL prior to any conversations with the research team to ensure people can read it first, think of questions and discussion with others. During the conversation, use simple and straightforward language, including speaking slowly, clearly and in short sentences. Be proactive to ask if people understand the information being presented and have any questions throughout the conversation. Avoid asking too many or overly complicated research or consent questions. Phrase questions in a way that allows for a simple answer—questions with a ‘yes’ or ‘no’ answer are easier to answer. Stick to one idea at a time. Giving someone a choice is important, but too many options can be confusing and frustrating. Offer culturally appropriate support, avoiding assumptions and recognise the impact that stigma and/or trauma may have on individual willingness to respond or participate	National Voices summary, The Adventurers, Lawnmowers
Wider support	Offer wider support as well as a PIL. People need more than just information to be motivated to become more actively involved in decisions. To be truly effective, information needs to be provided in a context of more active encouragement, education and support	National Voices summary

### Formatting

Text should be left-aligned, and bullet points, lists or sections used to break up the text. Use colour, considering readability in the selection of colours. Format text and images into two columns, with images in the left column. Headings should be easily distinguishable from the body of the text, short, and structured as questions. Print PILs on low-to-no gloss paper in an appropriate-sized booklet format and make it clear if readers need to turn overleaf. Select wider typefaces in a large font size (which can be increased if needed), avoid underlining, using text that is all in capitals and italics, and only use bold type face in the main text for emphasis. Include space without text (whitespace) to help with readability.

### Information presentation

Using a layered/tiered approach can help structure the provision of accessible information. Information needs to be presented in a logical order, with key/important messages first. Focus on one message at a time with related information grouped together and consider including summaries. Keep the volume of information short and avoid repetition or unnecessary information; the focus should be on the provision of enough information for people to make an informed decision about participation. Use appropriate, familiar and inclusive images that are relevant to the trial, that explain the text, support the main messages of the PIL, and/or explain a difficult concept. Limit the use of statistics, and if they are used consider how best to convey these to readers, including the use of image and analogies to explain numbers and statistical concepts.

### Writing style

Work in partnership with communities to co-produce accessible PILs and ensure a writing style that will be accessible to all readers. Use familiar, appropriate and inclusive phrasing, analogies and terminology. Use clear and familiar plain language, written in a conversational and narrative style, demonstrating respect and value for the readers. Ensure the PIL is written at an appropriate reading age and test readability with online readability tools (for example https://www.thefirstword.co.uk/readabilitytest/, https://goodcalculators.com/flesch-kincaid-calculator/ or https://www.thewriter.com/tools/readability) and/or user testing. Avoid jargon, assumptions and patronising language. Minimise the use of abbreviations and acronyms and where they are necessary explain them immediately and clearly. Write from the reader’s perspective; approach the information to be provided from the point of view of what the reader wants and needs to know, rather than what the researchers think they need to convey. Use an active voice and short words, sentences and paragraphs. Provide context for new information and ensure consistency throughout.

### Content

If using a front page, provide a concise overview of the trial and avoid using too many logos. Explain the purpose of clinical trials and the purpose of the trial, emphasising that participation is voluntary and encourage readers to discuss with other people before deciding about participation. Describe the importance of research participation and clearly convey the existing uncertainty that underpins the need for the trial. Clearly describe eligibility criteria, treatment allocation, the treatment(s) and standard care, and any treatment side effects. Explain study processes, including how data will be collected, handled and stored. Describe the advantages and disadvantages of participation, any incentives for participation and withdrawal processes. Provide an ethics statement and contact information for the research team and an independent advisor/advocate.

### Accessibility

Translate the accessible PIL into different languages and ensure communication and interpretation support is available for written and verbal information. Provide information in multiple formats e.g. braille, large print, plain text, audio, video format with voiceover/subtitles. Ensure the verbal information that is provided in any conversations with potential participants is clear, simple and culturally appropriate and offers wider support as well as information.

## Discussion

The MAPLE project has developed a comprehensive framework of recommendations to guide researchers in the development of accessible PILs for clinical trials. A previous literature review identified recommendations for accessible clinical research PILs and conducted work with stakeholders to support the development of patient-facing documents through expert consensus [24]. These were included in our work and extended further through working in partnership with marginalised community groups, patients and charities to ensure that a diverse range of voices and experiences informed the framework. These recommendations aim to support researchers to develop better study information to reduce English language literacy as a barrier to participation in clinical trials.

It is important to consider the strengths and limitations of this work within a broader context. A literature review was conducted rather than a systematic review, as the aim of the review was to gain an understanding of what is already known on the topic to inform the development of the recommendations framework rather than provide a definitive answer to a clinical question. While this approach was appropriate to the aim of this project, it may have led to relevant sources not being included as database searches were limited to MEDLINE and Embase. A key strength of this project was that it was conducted in partnership with diverse groups of people who may experience English language literacy as a barrier to research participation in different ways. Building trust and relationships and understanding preferred ways of working is an essential first stage to inclusive involvement in health research [41]. Based on the preferences of the groups and charities involved in this project, the discussions were not audio-recorded to ensure people felt comfortable and safe to contribute. Identifying and sharing example accessible PILs was a useful tool for promoting discussion, as many members of the community groups had not been approached about participating in a research project before and therefore had not seen a PIL. Discussions focussed on how the example accessible PILs could be improved and the reasons for the recommendations given were not explored due to time constraints. We acknowledged that the project likely did not encapsulate the views of all groups of people who may experience English language literacy as a barrier to research participation. We did not collect information on the protected characteristics of the people involved in the meetings and therefore are unable to comment on the full diversity of those involved; however, from working with community group members, they are likely at the intersection of multiple factors of marginalisation, for example, language, older age, digital exclusion, carers, multiple health conditions and disability. Also while some of our recommendations will apply across phase 1, phase 2 and phase 3 clinical trials, further work is needed to develop recommendations specifically focussed on developing accessible information for first-in-human clinical trials as the information requirements differ from later phase trials.

The MAPLE recommendations provide a preliminary framework to support the development of accessible PILs for clinical trials, however further work is needed to facilitate the use of the recommendations. Regulatory authorities are perceived by researchers as the largest barrier to the use of accessible PILs due to the need to meet regulatory and legal requirements [71]. However, regulatory authorities are often supportive of improving the accessibility of research, for example, the NHS Health Research Authority recommends a layered approach to information provision [72] and has developed principles and hallmarks of people-centred clinical research which includes ensuring that clinical research is accessible and communicated well to people [73]. There are also challenges in addressing all the recommendations regarding the content of an accessible PIL while ensuring readability and keeping the PIL short. Co-production work with patients and communities supports researchers to develop accessible PILs. However, this potentially poses a high and unsustainable burden to communities to be involved in creating new accessible PILs for each clinical trial, and further work is needed to support the creation of co-developed generic content that can then be tailored to individual clinical trials. Finally, investment from health research funders is needed to ensure that the additional funding required to implement measures to facilitate accessibility is available and prioritised within grant applications.

An additional important finding from this project was that a written PIL is only one method of providing information and needs to be supplemented with alternative formats to improve accessibility, for example, videos with subtitles provided in multiple languages and interpretation at research sites. The verbal information provided by research staff and clinicians about a trial is of paramount importance and needs to be culturally appropriate and clear, and support provided to enable people from marginalised backgrounds to participate in research. System-level change in approaches to recruitment is needed to improve the accessibility of clinical research, with researchers, patients, members of the public and regulatory authorities working in partnership to provide better information.

The development of the MAPLE recommendations can support researchers to develop accessible PILs for clinical trials and contribute towards addressing equity in health research participation. Our recommendations contribute to a growing body of work that aims to improve accessibility in clinical trials. However, providing more accessible and inclusive information is only one part of the complex array of barriers to research participation which need to be addressed. Historically, researchers have misconstrued that people from marginalised communities are unwilling to participate in research [1], when the reality is that people from these communities are not invited to participate, with barriers imposed by researchers [74]. Barriers to inclusive trials are surmountable and there is a need for investment to action systemic changes across health research to improve inclusivity and minimise the perpetuation of existing health inequalities [1, 6]. Evidence-based strategies and enablers to inclusive trials include cultural competency training, community partnerships, personalised approach, multilingual materials and staff, communication-specific strategies, increasing understanding and trust and tackling logistical barriers [5, 7, 75]. A multi-faceted approach, with investment from all stakeholders, is required to action and implement widespread changes to clinical trials and improve inclusion.

## Supplementary Information

Supplementary Material 1: Supplementary Table 1: Scoping review’ search strategy as applied to Embase. Supplementary Table 2: Summary of included articles identified in the literature view. Supplementary Table 3: Extracted recommendations from included sources, grouped by topic.

## Data Availability

The datasets generated and/or analysed during the current study are not publicly available as they comprise notes taken during community meetings and we do not have permission from community group members to share these notes.

## Abbreviations

INCLUDE: Innovations in Clinical Trial Design and Delivery for the underserved
MAPLE study: Making trials more Accessible through better Patient information Leaflets
NIHR: National Institute for Health Research
PCIE: Public and community involvement and engagement
PEP-R group: Patient Experience Partnership in Research
PIL: Patient information leaflet

## References

[1] Bibbins-Domingo K, Helman A, Dzau VJ. The imperative for diversity and inclusion in clinical trials and health research participation. JAMA. 2022;327:2283–4. 10.1001/jama.2022.9083. 2022/05/18. 35579885 10.1001/jama.2022.9083

[2] Treweek S, Banister K, Bower P, et al. Developing the INCLUDE Ethnicity Framework-a tool to help trialists design trials that better reflect the communities they serve. Trials. 2021;22:337. 10.1186/s13063-021-05276-8. 2021/05/12. 33971916 10.1186/s13063-021-05276-8 PMC8108025

[3] Herieka H, Babalis D, Tzala E, et al. How inclusive were UK-based randomised controlled trials of COVID-19 vaccines? A systematic review investigating enrolment of Black adults and adult ethnic minorities. Trials. 2024;25:255. 10.1186/s13063-024-08054-4. 2024/04/12. 38605411 10.1186/s13063-024-08054-4 PMC11010339

[4] Witham MD, Anderson E, Carroll C, et al. Developing a roadmap to improve trial delivery for under-served groups: results from a UK multi-stakeholder process. Trials. 2020;21:694. 10.1186/s13063-020-04613-7. 2020/08/03. 32738919 10.1186/s13063-020-04613-7 PMC7395975

[5] Dawson S, Banister K, Biggs K, et al. Trial Forge Guidance 3: randomised trials and how to recruit and retain individuals from ethnic minority groups-practical guidance to support better practice. Trials. 2022;23:672. 10.1186/s13063-022-06553-w. 2022/08/18. 35978338 10.1186/s13063-022-06553-w PMC9383663

[6] Corneli A, Hanlen-Rosado E, McKenna K, et al. Enhancing Diversity and Inclusion in Clinical Trials. Clin Pharmacol Ther. 2023;113:489–99. 10.1002/cpt.2819. 2023/01/12. 36628990 10.1002/cpt.2819

[7] Bodicoat DH, Routen AC, Willis A, et al. Promoting inclusion in clinical trials-a rapid review of the literature and recommendations for action. Trials. 2021;22:880. 10.1186/s13063-021-05849-7. 2021/12/06. 34863265 10.1186/s13063-021-05849-7 PMC8643184

[8] National Literacy Trust. Adult literacy rates in the UK. https://literacytrust.org.uk/parents-and-families/adult-literacy/, (accessed 11/04/2024).

[9] O’Sullivan L, Sukumar P, Crowley R, et al. Readability and understandability of clinical research patient information leaflets and consent forms in Ireland and the UK: a retrospective quantitative analysis. BMJ Open. 2020;10:e037994. 10.1136/bmjopen-2020-037994. Article 2020/09/05. 32883734 10.1136/bmjopen-2020-037994 PMC7473620

[10] Symons T, Davis JS. Creating concise and readable patient information sheets for interventional studies in Australia: are we there yet? Trials. 2022;23:8. 10.1186/s13063-022-06712-z. Article. 36131293 10.1186/s13063-022-06712-z PMC9490706

[11] Foe G, Larson EL. Reading Level and Comprehension of Research Consent Forms: An Integrative Review. J Empir Res Hum Res Ethics. 2016;11:31–46. 10.1177/1556264616637483. Review 2016/04/24. 27106889 10.1177/1556264616637483

[12] Santel F, Bah I, Kim K, et al. Assessing readability and comprehension of informed consent materials for medical device research: a survey of informed consents from FDA’s center for devices and radiological health. Contemp Clin Trials. 2019;85:8. 10.1016/j.cct.2019.105831. Article. 10.1016/j.cct.2019.105831 31445173

[13] Emanuel EJ, Boyle CW. Assessment of length and readability of informed consent documents for COVID-19 vaccine trials. JAMA Netw Open. 2021;4:5. 10.1001/jamanetworkopen.2021.10843. Article. 10.1001/jamanetworkopen.2021.10843 PMC8082317 33909052

[14] Bull S, Cheah PY, Lwin KM, et al. Consent and community engagement in diverse research contexts: reviewing and developing research and practice. J Empir Res Hum Res Ethics. 2013;8:1–18. 10.1525/jer.2013.8.4.1. Article. 10.1525/jer.2013.8.4.1 PMC4836561 24169417

[15] Benatar JR, Mortimer J, Stretton M, Stewart RAH. A booklet on participants’ rights to improve consent for clinical research: a randomized trial. PLoS One. 2012;7:7. 10.1371/journal.pone.0047023. Article. 10.1371/journal.pone.0047023 PMC3477160 23094034

[16] Nishimura A, Carey J, Erwin PJ, et al. Improving understanding in the research informed consent process: a systematic review of 54 interventions tested in randomized control trials. BMC Med Ethics. 2013;14:15. 10.1186/1472-6939-14-28. Review. 23879694 10.1186/1472-6939-14-28 PMC3733934

[17] Bader M, Zheng L, Rao D, et al. Towards a more patient-centered clinical trial process: A systematic review of interventions incorporating health literacy best practices. Contemp Clin Trials. 2022;116:106733. 10.1016/j.cct.2022.106733. Review 2022/03/19. 35301134 10.1016/j.cct.2022.106733 PMC9196949

[18] NHS England. Guide to making information accessible for people with a learning disability. LearningDisabilityAccessCommsGuidance.pdf (england.nhs.uk. 2018. https://www.england.nhs.uk/wp-content/uploads/2018/06/LearningDisabilityAccessCommsGuidance.pdf.

[19] UK Standards for Public Involvement. Definitions used in the Standards.https://sites.google.com/nihr.ac.uk/pi-standards/standards/definitions*. *Accessed 16th August 2024.

[20] National Institute for Health research. Briefing notes for researchers - public involvement in NHS, health and social care research.https://www.nihr.ac.uk/documents/briefing-notes-for-researchers-public-involvement-in-nhs-health-and-social-care-research/27371*. *Accessed 16th August 2024.

[21] Lefebvre C, Glanville J, Briscoe S, Featherstone R, Littlewood A, Metzendorf M-I, Noel-Storr A, Paynter R, Rader T, Thomas J, Wieland LS. Chapter 4: Searching for and selecting studies. In: Higgins JPT, Thomas J, Chandler J, Cumpston M, Li T, Page MJ, Welch VA (editors). Cochrane Handbook for Systematic Reviews of Interventions version 6.4 (updated October 2023). Cochrane. 2023. Available from www.training.cochrane.org/handbook.

[22] Sustersic M, Gauchet A, Foote A, Bosson JL. How best to use and evaluate Patient Information Leaflets given during a consultation: a systematic review of literature reviews. Health Exp Int J Public Particip Health Care Health Pol. 2017;20:531–42. 10.1111/hex.12487. 2016/09/28. 10.1111/hex.12487 PMC5512995 27669682

[23] Gillies K, Huang W, Skea Z, et al. Patient information leaflets (PILs) for UK randomised controlled trials: a feasibility study exploring whether they contain information to support decision making about trial participation. Trials. 2014;15:62. 10.1186/1745-6215-15-62. 2014/02/20. 24548781 10.1186/1745-6215-15-62 PMC3936815

[24] Coleman E, O’Sullivan L, Crowley R, et al. Preparing accessible and understandable clinical research participant information leaflets and consent forms: a set of guidelines from an expert consensus conference. Res Involv Engagem. 2021;7:31. 10.1186/s40900-021-00265-2. 2021/05/20. 34006326 10.1186/s40900-021-00265-2 PMC8130271

[25] Medina-Cordoba M, Cadavid S, Perez-Acosta AM, Amaya-Giraldo V. Factors that facilitate and hinder the comprehension of Patient Information Leaflets (PILs): a brief scoping review. Front Pharmacol. 2021;12:740334. 10.3389/fphar.2021.740334. Mini Review 2021/12/04. 34858174 10.3389/fphar.2021.740334 PMC8631714

[26] Brierley G, Richardson R, Torgerson DJ. Using short information leaflets as recruitment tools did not improve recruitment: a randomized controlled trial. J Clin Epidemiol. 2012;65:147–54. 10.1016/j.jclinepi.2011.06.005. 2011/09/06. 21889304 10.1016/j.jclinepi.2011.06.005

[27] Hilton P, Buckley BS, McColl E, et al. Understanding variations in patient screening and recruitment in a multicentre pilot randomised controlled trial: a vignette-based study. Trials. 2016;17:522. 10.1186/s13063-016-1652-2. 2016/10/27. 27782847 10.1186/s13063-016-1652-2 PMC5080689

[28] Flory J, Emanuel E. Interventions to improve research participants’ understanding in informed consent for research: a systematic review. JAMA. 2004;292:1593–601. 10.1001/jama.292.13.1593. Review 2004/10/07. 15467062 10.1001/jama.292.13.1593

[29] Breese PE, Burman WJ, Goldberg S, Weis SE. Education level, primary language, and comprehension of the informed consent process. J Empir Res Hum Res Ethics. 2007;2:69–79. 10.1525/jer.2007.2.4.69. Article 2007/12/01. 19385809 10.1525/jer.2007.2.4.69

[30] Muzanyi G, Sekitoleko I, Johnson JL, et al. Level of education and preferred language of informed consent for clinical research in a multi-lingual community. Afr Health Sci. 2020;20:955–9. 10.4314/ahs.v20i2.51. Article 2020/11/10. 33163064 10.4314/ahs.v20i2.51 PMC7609127

[31] Baiden F, Akazili J, Chatio S, et al. Should consent forms used in clinical trials be translated into the local dialects? A survey among past participants in rural Ghana. Clin Trials. 2016;13:234–9. 10.1177/1740774515609290. Article 2015/10/11. 26452387 10.1177/1740774515609290

[32] Bonevski B, Randell M, Paul C, et al. Reaching the hard-to-reach: a systematic review of strategies for improving health and medical research with socially disadvantaged groups. BMC Med Res Methodol. 2014;14:42. 10.1186/1471-2288-14-42. Article 2014/03/29. 24669751 10.1186/1471-2288-14-42 PMC3974746

[33] Busisiwe N, Seeley J, Strode A, Parker M. Beyond translations, perspectives for researchers to consider to enhance comprehension during consent processes for health research in sub-saharan Africa: a scoping review. BMC Med Ethics. 2023;24:43. 10.1186/s12910-023-00920-1. Review 2023/06/22. 37344810 10.1186/s12910-023-00920-1 PMC10286482

[34] Cohn E, Larson E. Improving participant comprehension in the informed consent process. J Nurs Scholarsh. 2007;39:273–80. 10.1111/j.1547-5069.2007.00180.x. Article 2007/09/01. 17760802 10.1111/j.1547-5069.2007.00180.x

[35] Cortes DE, Drainoni ML, Henault LE, Paasche-Orlow MK. How to achieve informed consent for research from Spanish-speaking individuals with low literacy: a qualitative report. J Health Commun. 2010;15(Suppl 2):172–82. 10.1080/10810730.2010.499990. Article 2010/09/29. 20845202 10.1080/10810730.2010.499990

[36] Hughson JA, Woodward-Kron R, Parker A, et al. A review of approaches to improve participation of culturally and linguistically diverse populations in clinical trials. Trials. 2016;17:263. 10.1186/s13063-016-1384-3. Review 2016/05/28. 27229153 10.1186/s13063-016-1384-3 PMC4880985

[37] Woodward-Kron R, Fraser C, Rashid H, et al. Perspectives of junior doctor intercultural clinical communication: Lessons for medical education. Focus Health Prof Educ. 2016;17:82–95. 10.11157/fohpe.v17i3.179. Article.

[38] Shiely F, Daly A. Trial lay summaries were not fit for purpose. J Clin Epidemiol. 2023;156:105–12. 10.1016/j.jclinepi.2023.02.023. Article 2023/03/04. 36868328 10.1016/j.jclinepi.2023.02.023

[39] Sudore RL, Landefeld CS, Williams BA, et al. Use of a modified informed consent process among vulnerable patients: a descriptive study. J Gen Intern Med. 2006;21:867–73. 10.1111/j.1525-1497.2006.00535.x. Article 2006/08/03. 16881949 10.1111/j.1525-1497.2006.00535.x PMC1831581

[40] Yu Z, Kowalkowski J, Roll AE, Lor M. Engaging underrepresented communities in health research: lessons learned. West J Nurs Res. 2021;43:915–23. 10.1177/0193945920987999. Article 2021/01/16. 33448251 10.1177/0193945920987999

[41] Jameson C, Haq Z, Musse S, et al. Inclusive approaches to involvement of community groups in health research: the co-produced CHICO guidance. Res Involv Engagem. 2023;9:76. 10.1186/s40900-023-00492-9. 13063_2024_8471. 37679854 10.1186/s40900-023-00492-9 PMC10486022

[42] National Voices. English literacy as a barrier to participation in clinical trials.https://s42139.pcdn.co/wp-content/uploads/English-literacy-as-a-barrier-to-participation-in-clinical-trials-MAPLE-REND-Project.pdf. 2024.

[43] Head KJ, Hartsock JA, Bakas T, et al. Development of written materials for participants in an alzheimer’s disease and related dementias screening trial. J Patient Exp. 2022;9:7. 10.1177/23743735221092573. Article. 10.1177/23743735221092573 PMC9009139 35434299

[44] Atwere P, McIntyre L, Carroll K, et al. Informed consent documents used in critical care trials often do not implement recommendations. Crit Care Med. 2018;46:E111–7. 10.1097/ccm.0000000000002815. Article. 29088004 10.1097/CCM.0000000000002815

[45] Dellson P, Nilbert M, Carlsson C. Patient representatives’ views on patient information in clinical cancer trials. BMC Health Serv Res. 2016;16:5. 10.1186/s12913-016-1272-2. Article. 26831330 10.1186/s12913-016-1272-2 PMC4736467

[46] Addissie A, Abay S, Feleke Y, et al. Cluster randomized trial assessing the effects of rapid ethical assessment on informed consent comprehension in a low-resource setting. BMC Med Ethics. 2016;17:12. 10.1186/s12910-016-0127-z. Article. 27406063 10.1186/s12910-016-0127-z PMC4943010

[47] Eeckhout D, Aelbrecht K, Van der Straeten C. Informed consent: research staff’s perspectives and practical recommendations to improve research staff-participant communication. J Empir Res Hum Res Ethics. 2023;18:3–12. 10.1177/15562646221146043. Article. 36562147 10.1177/15562646221146043

[48] Spellecy R, Tarima S, Denzen E, et al. Easy-to-read informed consent form for hematopoietic cell transplantation clinical trials: results from the blood and marrow transplant clinical trials network 1205 study. Biol Blood Marrow Transpl. 2018;24:2145–51. 10.1016/j.bbmt.2018.04.014. Article. 10.1016/j.bbmt.2018.04.014 PMC6193865 29679770

[49] Simonds VW, Garroutte EM, Buchwald D. Health literacy and informed consent materials: designed for documentation, not comprehension of health research. J Health Commun. 2017;22:682–91. 10.1080/10810730.2017.1341565. Article. 28759329 10.1080/10810730.2017.1341565 PMC6155979

[50] Lentz J, Kennett M, Perlmutter J, Forrest A. Paving the way to a more effective informed consent process: Recommendations from the Clinical Trials Transformation Initiative. Contemp Clin Trials. 2016;49:65–9. 10.1016/j.cct.2016.06.005. Article. 27327780 10.1016/j.cct.2016.06.005

[51] Lorell BH, Mikita JS, Anderson A, et al. Informed consent in clinical research: Consensus recommendations for reform identified by an expert interview panel. Clin Trials. 2015;12:692–5. 10.1177/1740774515594362. Article. 26178662 10.1177/1740774515594362 PMC4657389

[52] Kass NE, Taylor HA, Ali J, et al. A pilot study of simple interventions to improve informed consent in clinical research: Feasibility, approach, and results. Clin Trials. 2015;12:54–66. 10.1177/1740774514560831. Article. 25475879 10.1177/1740774514560831 PMC4344898

[53] Quinn SC, Garza MA, Butler J, et al. Improving informed consent with minority participants: results from researcher and community surveys. J Empir Res Hum Res Ethics. 2012;7:44–55. 10.1525/jer.2012.7.5.44. Article. 23324203 10.1525/jer.2012.7.5.44 PMC3685140

[54] Denzen EM, Santibáñez MEB, Moore H, et al. Easy-to-read informed consent forms for hematopoietic cell transplantation clinical trials. Biol Blood Marrow Transplant. 2012;18:183–9. 10.1016/j.bbmt.2011.07.022. Review. 21806948 10.1016/j.bbmt.2011.07.022 PMC3242929

[55] Dellson P, Nilbert M, Bendahl PO, et al. Towards optimised information about clinical trials; identification and validation of key issues in collaboration with cancer patient advocates. Eur J Cancer Care. 2011;20:445–54. 10.1111/j.1365-2354.2010.01207.x. Article. 10.1111/j.1365-2354.2010.01207.x 20738392

[56] Jefford M, Moore R. Improvement of informed consent and the quality of consent documents. Lancet Oncol. 2008;9:485–93. 10.1016/s1470-2045(08)70128-1. Article. 18452859 10.1016/S1470-2045(08)70128-1

[57] Adams V, Miller S, Craig S, et al. Informed consent in cross-cultural perspective: Clinical research in the Tibetan Autonomous Region. PRC Cult Med Psychiatr. 2007;31:445–72. 10.1007/s11013-007-9070-2. Article. 10.1007/s11013-007-9070-2 17968637

[58] Corneli AL, Bentley ME, Sorenson JR, et al. Using formative research to develop a context-specific approach to informed consent for clinical trials. J Empir Res Hum Res Ethics. 2006;1:45–60. 10.1525/jer.2006.1.4.45. Article. 19385837 10.1525/jer.2006.1.4.45 PMC3140046

[59] Simonds VW, Buchwald D. Too Dense and Too Detailed: Evaluation of the Health Literacy Attributes of an Informed Consent Document. J Racial Ethn Health Disparities. 2020;7:327–35. 10.1007/s40615-019-00661-1. Article. 31823337 10.1007/s40615-019-00661-1 PMC7067617

[60] Brockhoven F, Raphael M, Currier J, et al. REPRESENT recommendations: improving inclusion and trust in cancer early detection research. Br J Cancer. 2023;129:1195–208. 10.1038/s41416-023-02414-8. Article. 37689805 10.1038/s41416-023-02414-8 PMC10575902

[61] Cunningham-Erves J, Kusnoor SV, Villalta-Gil V, et al. Development and pilot implementation of guidelines for culturally tailored research recruitment materials for African Americans and Latinos. BMC Med Res Methodol. 2022;22:14. 10.1186/s12874-022-01724-4. Article. 36153481 10.1186/s12874-022-01724-4 PMC9508728

[62] Jilka S, Hudson G, Jansli SM, et al. How to make study documents clear and relevant: the impact of patient involvement. BJPsych Open. 2021;7:8. 10.1192/bjo.2021.1040. Article.

[63] Burks AC, Keim-Malpass J. Health literacy and informed consent for clinical trials: a systematic review and implications for nurses. Nursing. 2019;9:31–40. 10.2147/nrr.S207497. Review.

[64] Mayers SA, Cook SK, Rantala C, et al. The RIC Recruitment & Retention Materials Toolkit - a resource for developing community-informed study materials. J Clin Transl Sci. 2023;7:e182.10.1017/cts.2023.607 37706001 10.1017/cts.2023.607 PMC10495822

[65] Tait AR, Voepel-Lewis T, Malviya S, Philipson SJ. Improving the readability and processability of a pediatric informed consent document: effects on parents’ understanding. Arch Pediatr Adolesc Med. 2005;159:347–52. 10.1001/archpedi.159.4.347. 2005/04/06. 15809387 10.1001/archpedi.159.4.347

[66] Beasant L, Realpe A, Douglas S, et al. Autistic adults' views on the design and processes within randomised controlled trials: The APRiCoT study. Autism 2023: 13623613231202432. 10.1177/13623613231202432. 10.1177/13623613231202432 PMC11134970 37882480

[67] Tait AR, Voepel-Lewis T, Nair VN, et al. Informing the uninformed: optimizing the consent message using a fractional factorial design. JAMA Pediatr. 2013;167:640–6. 10.1001/jamapediatrics.2013.1385. 2013/05/24. 23700028 10.1001/jamapediatrics.2013.1385 PMC3700595

[68] US Department of Health and Human Services Centres for Disease Control and Prevention. Simply Put: A guide for creating easy-to-understand materials. https://stacks.cdc.gov/view/cdc/11938. July 2010, Third Edition.

[69] Health Research Authority. Consent and Participant Information Guidance. https://www.hra-decisiontools.org.uk/consent/style.html, (accessed 5th January 2024).

[70] Dunman M. Producing patient information : how to research, develop and produce effective information sources London: King's Fund; 2003 [Available from: https://archive.kingsfund.org.uk/concern/published_works/000030954?locale=en#?cv=8&xywh=191,168,1219,696.

[71] Solomon ED, Mozersky J, Wroblewski MP, et al. Understanding the use of optimal formatting and plain language when presenting key information in clinical trials. J Empir Res Hum Res Ethics. 2022;17:177–92. 10.1177/15562646211037546. 2021/08/20. 34410175 10.1177/15562646211037546 PMC8712347

[72] Health Research Authority. Applying a proportionate approach to the process of seeking consent. https://s3.eu-west-2.amazonaws.com/www.hra.nhs.uk/media/documents/Proportionate_approach_to_seeking_consent_HRA_Guidance.pdf. 2019.

[73] NHS Health Research Authority. People-Centred Clinical Research. https://www.hra.nhs.uk/planning-and-improving-research/best-practice/people-centred-clinical-research/, (2024, accessed 20th May 2024).

[74] Brijnath B, Muoio R, Feldman P, et al. “We are not invited”: Australian focus group results on how to improve ethnic diversity in trials. J Clin Epidemiol. 2024;170:111366. 10.1016/j.jclinepi.2024.111366 2024/04/18. 38631530 10.1016/j.jclinepi.2024.111366

[75] Goodwin VA, Low MSA, Quinn TJ, et al. Including older people in health and social care research: best practice recommendations based on the INCLUDE framework. Age Ageing. 2023;52:afad082. 10.1093/ageing/afad082. 2023/06/01. 37261448 10.1093/ageing/afad082 PMC10234283

