# Supplementary material for: Recommendations for developing accessible patient information leaflets for clinical trials to address English language literacy as a barrier to research participation
Source: Trials. 2024 Sep 27;25:624. doi: 10.1186/s13063-024-08471-5 (PMC11430508; doi:10.1186/s13063-024-08471-5)
Supplement: Supplementary file 1 — Supplementary Material 1: Supplementary Table 1: Scoping review’ search strategy as applied to Embase. Supplementary Table 2: Summary of included articles identified in the literature view. Supplementary Table 3: Extracted recommendations from included sources, grouped by topic. [file 13063_2024_8471_MOESM1_ESM.docx]

**Supplementary Table 1: Scoping review search strategy as applied to Embase**

1., Randomized controlled trial/ or Controlled clinical study/ or random$.ti,ab. or randomization/ or intermethod comparison/ or placebo.ti,ab. or (compare or compared or comparison).ti. or ((evaluated or evaluate or evaluating or assessed or assess) and (compare or compared or comparing or comparison)).ab. or (open adj label).ti,ab. or ((double or single or doubly or singly) adj (blind or blinded or blindly)).ti,ab. or double blind procedure/ or parallel group$1.ti,ab. or (crossover or cross over).ti,ab. or ((assign$ or match or matched or allocation) adj5 (alternate or group$1 or intervention$1 or patient$1 or subject$1 or participant$1)).ti,ab. or (assigned or allocated).ti,ab. or (controlled adj7 (study or design or trial)).ti,ab. or (volunteer or volunteers).ti,ab. or human experiment/ or trial.ti.

2., ((Handout or Leaflet or Booklet or Pamphlet or Flyer or Folder or Brochure or Written patient information) and patient).tw.

3., 1 and 2

**Supplementary Table 2: Summary of included articles identified in the literature view**

| **Authors** | **Country** | **Participants** | **Study aim** | **Study methods** |
| --- | --- | --- | --- | --- |
| Adams et al, 2007^1^ | Tibet | Pregnant women and hospital providers | To establish a culturally appropriate Informed Consent process for biomedical research in the Tibet Autonomous Region in the People’s Republic of China | Qualitative interviews and focus groups, piloting of informed consent documents, survey |
| Addissie et al, 2016^2^ | Ethiopia | Pregnant women | To assess the effects of Rapid Ethical Assessment on comprehension,  retention and quality of the informed consent process | Measurement of informed consent comprehension levels and quality of the consent process within a RCT using the Modular Informed Consent Comprehension Assessment and Quality of  Informed Consent process assessment tools |
| Atwere et al, 2017^3^ | Canada | Not applicable | To examine how well RCTs implement existing recommendations for addressing critical care-specific consent issues | Systematic review of the literature and review of informed consent documents for RCTs. |
| Beasant et al, 2023^4^ | UK | Autistic people | To understand the barriers and facilitators for recruiting autistic people  to RCTs involving medications. | Qualitative interviews |
| Benatar et al, 2012^5^ | USA | Hospital inpatients | To evaluate whether a simple booklet which is relevant to all research studies improved the understanding of rights needed for people to provide informed consent for a RCT | Comparison of comprehension of information after provision of a standard informed consent form (ICF), short ICF and booklet or a simplified ICF and booklet. |
| Bodicoat et al, 2021^6^ | UK | Not applicable | To evaluate barriers relating to inclusion of under-served groups in research, and evidence of approaches that are effective in overcoming these. | Systematic review |
| Bonevski et al, 2014^7^ | Australia | Not applicable | To review barriers to sampling, recruitment, participation,  and retention of members of socioeconomically disadvantaged groups in health research and strategies for  increasing the amount of health research conducted with socially disadvantaged groups | Systematic review |
| Brockhoven et al, 2023^8^ | USA and UK | Community representatives  and public contributors, researchers, research managers, coordinators, and research funding  representatives | Development recommendations to build relations of trust and include minoritized groups in early detection cancer research | Stakeholder consensus work, informed by a systematic review and two  ethnographic case studies |
| Burks et al, 2019^9^ | USA | Not applicable | Explore the literature related to health literacy and the informed consent process for clinical trials | Systematic review |
| Cohn and Larson, 2017^10^ | USA | Not applicable | To critically analyse studies about participants’ comprehension of informed consent in clinical research and to identify promising intervention strategies. | Integrative review of literature |
| Coleman et al, 2021^11^ | Ireland | Lay and expert Research Ethics Committee members, patient advocate, researchers, plain English editor and Data Protection Officer | To propose and agree a set of guidelines for academic researchers and sponsors for preparing accessible and understandable PILs/ICFs. | Literature review, stakeholder consensus work |
| Corneli et al, 2006^12^ | USA and Malawi | Malawi women infected with HIV, teachers and research nurses, and community leaders and members of the community advisory board. | To explore the community’s  understanding of medical research in Malawi and how to explain research through local terms and meanings. | Qualitative interviews and focus groups |
| Cunningham-Erves et al, 2022^13^ | USA | African Americans and Latinos | To develop and pilot testing of recruitment material guidelines used to culturally tailor clinical trial recruitment materials targeting African Americans and Latinos | Literature review, focus groups, pilot study |
| Dellson et al, 2016^14^ | Sweden and Denmark | Patients from Swedish patient associations for gastrointestinal diseases | To explore patient representatives’ views and perceptions on the written trial information used in clinical cancer trials | Focus groups |
| Dellson et al, 2011^15^ | Sweden and Denmark | Breast cancer patient advocates | To study patients’ opinions about the written information used in three clinical trials for breast cancer | Focus groups |
| Denzen et al, 2012^16^ | USA | Members of the Blood and Marrow Transplant Clinical Trials Network | To develop recommendations for developing easy-to-read ICFs for clinical trials | Literature search, creation of easy-to-read ICF, |
| Eeckhout et al, 2023^17^ | Belgium | Research staff | To provide make practical recommendations to improve communication between research staff and participants | Qualitative interviews |
| Flory and Emanuel, 2004^18^ | USA | Not applicable | To review research on interventions to improve research participants’ understanding  of information disclosed in the informed consent process | Systematic review |
| Foe and Larson, 2016^19^ | USA | Not applicable | To assess and address the extent of the problem regarding the level of literacy of consent forms | Integrative literature review |
| Head et al, 2022^20^ | USA | Not applicable | To designed theoretically-based written participant communication materials to enhance the participant experience and provide important clinical information  during the trial | Case study of development of written participant communication materials used in a trial with people with Alzheimer’s disease |
| Hughson et al, 2016^21^ | Australia | Not applicable | To examine the complex issue of recruiting  culturally and linguistically diverse older people to medical research and to examine responses to these issues | Literature review |
| Jefford and Moore, 2008^22^ | Australia | Not applicable | To analyse the written consent form,  particularly in the context of clinical research, and the discussions that take place between clinician or investigator and patient, and development recommendations. | Literature review |
| Jilka et al, 2021^23^ | UK | Not applicable | To investigate the impact of the Feasibility and Support to Timely Recruitment for Research (FAST-R) service and provide researchers with guidelines to improve study documents | Mixed-methods study auditing study documents submitted to FAST-R before and after review |
| Kass et al, 2015^24^ | USA | Participants in eight ongoing clinical trials | To examine the feasibility of testing two consent interventions in actual studies and also to measure effectiveness of interventions in improving understanding of trials. | Assigned of RCT participants to one of three study arms involving different informed consent procedures (one control and two intervention), with use of closed and open ended questions to assess patient understanding |
| Lentz et al, 2016^25^ | USA | Representatives from academia, nonprofit  organisations, government agencies, ethics panels, industry, independent  consulting companies, health systems, patient representatives, law firms, and professional societies. | To develop recommendations for a more effective informed consent process | Literature review, expert interviews and stakeholder meeting |
| Lorell et al, 2015^26^ | USA | Experienced observers of the informed consent process | To examine the recommendations of experts in the informed consent process | Qualitative interviews |
| Mayers et al, 2023^27^ | USA | Not applicable | To develop a Recruitment & Retention Materials Content and Design Toolkit | Toolkit development, including feedback from two community stakeholder groups |
| Quinn et al, 2012^28^ | USA | Researchers and African American and Latino participants | To examine the process of informed consent from the perspective of researchers and African American and Latino community members | Survey |
| Simonds et al, 2017^29^ | USA | Not applicable | To develop recommendations for an improved consent process | Review of consent forms and associated Institutional Review Board websites |
| Simonds and Buckwald, 2020^30^ | USA | People of American Indian race | To explore American Indian research participants’ understanding and perceptions of an example consent document | Focus groups |
| Spellecy et al, 2018^31^ | USA | Patients approached about clinical trial participation | To evaluate an easy-read informed consent form | Randomised comparison of comprehension of an easy-read informed consent form and standard consent form |
| Tait et al, 2005^32^ | USA | Parents of children scheduled  for minor elective surgical procedures | To examine whether a consent document  modified to conform with guidelines for readability results in greater parental  understanding compared with a standard form. | Randomised comparison of standard consent form alone, standard consent form with verbal disclosure, modified form alone, and modified form with  verbal disclosure, with interviews to determine understanding of information |
| Tait et al, 2013^33^ | USA | Parents of children scheduled for elective surgery | To examine the effect of different communication strategies on parental  understanding of research information | Randomised comparison of different combinations of communication strategies for a hypothetical pain trial, with interviews to determine understanding of information |

**Supplementary table 3: Extracted recommendations from included sources, grouped by topic**

| **Article/Source** | **Extracted data on recommendations** |
| --- | --- |
| **Alignment** |  |
| Centres for Disease Control and Prevention Simply Put | Use right edge “ragged” or unjustified for the best readability. |
| Coleman et al, 2021 | Use left aligned text |
| Denzen et al, 2012 | Layout - left margins are justified and leave the right margins ragged |
| HRA and Cochrane collaboration | Use non-justified text. |
| **Break up text** | |
| Simply put - CDC | Avoid lengthy lists. Create short lists (3-7 items) with bullets, not commas. People with limited reading skills tend to forget items in longer lists. If you have a long list, break it into subheads. |
| Simply put - CDC | Break up text with bullets |
| Coleman et al, 2021 | Use bullet points or numbered lists, rather than long sentences with lists of items, but ideally use no more than 7 bullet points. |
| Dellson et al, 2016 | Use bullet points |
| Denzen et al, 2012 | Plain language - break up long sentences into bulleted lists |
| Foe et al, 2016 | Bullet points |
| Head et al, 2022 | numbered lists |
| HRA and Cochrane collaboration | Use bullets for lists. |
| Jefford et al, 2008 | Use of bullet points to break-up long explanations |
| Jilka et al, 2021 | Use bullets for lists |
| Kass et al, 2015 | Use bullet points |
| Lentz et al, 2016 | Incorporate lists |
| Simonds et al, 2017 | Use bulleted lists |
| Simonds et al, 2020 | Use of bullet points |
| Tait et al, 2005 | Use bullet points |
| **Colour** | |
| Simply put - CDC | Use dark letters on a light background. Light text on a dark background is harder to read. |
| Cohn et al, 2007 | Add colour |
| Dellson et al, 2016 | Use colours |
| Mayers et al, 2023 | Appropriate use of contrast and colour is stressed to ensure study materials are accessible and easy to read |
| **Columns** | |
| Simply put - CDC | Use columns. Columns with line lengths of 40 to 50 characters are easiest to read. |
| Coleman et al, 2021 | If columns are used, there should be enough separation between them to sufficiently separate the text. |
| Denzen et al, 2012 | Layout - use a two-column format |
| Spellecy et al, 2018 | Two column easy to read PIL was viewed favourably by clinical trial sites |
| Tait et al, 2005 | Use column format |
| **Headings** | |
| Simply put - CDC | For headings, use a font size at least 2 points larger than the main text size. |
| Simply put - CDC | Use headings and sub-headings to “chunk” text |
| Simply put - CDC | Questions can be successfully used as subheadings. |
| Simply put - CDC | Leave more space above headings and subheadings than below them |
| Coleman et al, 2021 | Headings should be used and should be easily distinguished from the body of the text, using bold type face, a larger type size or a different colour. Each heading of the same level should have the same distinguishable format for example, each section of the leaflet could be captioned by a larger heading, with each subsection within them having the same smaller subheading. The colours red or green should be avoided in the words of a heading |
| Coleman et al, 2021 | Use questions in section headings for example: “What are the risks of taking part in this study?” |
| Dellson et al, 2016 | Structured contents using headings and sections |
| Dellson et al, 2011 | Use headings followed by short passages |
| Dellson et al, 2011 | Use headings made up from questions |
| Denzen et al, 2012 | Layout - keep headers as close to text as possible |
| Denzen et al, 2012 | Organisation - use simple headers to break up text |
| HRA and Cochrane | Use short headings that stand out |
| HRA and Cochrane | A question and answer format is often effective. |
| Jilka et al, 2021 | Use headings |
| Simonds et al, 2017 | Titles, subtitles, and other headers help to clarify organization of text |
| Simonds et al, 2017 | Section headings should be in question format |
| Simonds et al, 2020 | Use headings |
| **Paper** | |
| Coleman et al, 2021 | Should be a booklet (but if dexterity an issue then should be A4 page) |
| Coleman et al, 2021 | Use low-to-no gloss paper |
| HRA and Cochrane | Consider the appropriate page size – it may be that A5, or another paper-size and layout would be more suitable than A4. |
| **Typeface** | |
| Bonevski et al, 2014 | Large font |
| Simply put - CDC | Use font sizes between 12 and 14 points. Anything less than 12 points can be too small to read for many audiences. Older people and people who have trouble reading or seeing may need larger print. |
| Simply put - CDC | For the body of the text, use fonts with serifs, like the one used in this line. Serif fonts are usually easier to read than sans-serif fonts. This is because the serif makes the individual letters more distinctive and easier for our brains to recognize quickly. Serifs are the little “feet” on letters. |
| Simply put - CDC | Do not use FANCY or script lettering |
| Simply put - CDC | Use both upper and lower case letters. Do not use ALL CAPS. ALL CAPS ARE HARD TO READ. |
| Simply put - CDC | Use grammatically correct punctuation. |
| Simply put - CDC | Use bold type to emphasize words or phrases. |
| Simply put - CDC | Limit the use of italics or underlining. They are hard to read. |
| Coleman et al, 2021 | Body of text should be at least type size 12. A soft copy of the PIL should be available so that the font size can be altered based on the needs of the trial participant, for example, increased for a visually impaired participant. Similarly, if a pdf document is provided to participants, text boxes should be tested to ensure they are readable by screen readers. |
| Coleman et al, 2021 | Use a sans serif font (for example: Arial, Verdana, Tahoma) |
| Coleman et al, 2021 | The main message should be emphasized with visual cues – larger font, headings etc. |
| Coleman et al, 2021 | Use uppercase and lowercase letters – lines of ‘All Capitals’ should be avoided. |
| Coleman et al, 2021 | Underlining should be avoided |
| Coleman et al, 2021 | Italics should be avoided |
| Denzen et al, 2012 | Typography - Sans-serif fonts are appropriate for section headers |
| Denzen et al, 2012 | Typography - use 11-13 point size |
| Denzen et al, 2012 | Typography - Serif fonts are preferred for text, avoid underlining or italicizing blocks of text or all capitals |
| Foe et al, 2016 | Use larger font |
| Head et al, 2022 | Use size 12 font |
| HRA and Cochrane | Use type as large as possible - size 16 font if you intend to recruit elderly subjects. |
| HRA and Cochrane | Use bold lower case for emphasis. |
| Jilka et al, 2021 | Use bold for emphasis |
| Mayers et al, 2023 | Size in 12-point font or larger |
| Mayers et al, 2023 | Arial font has very narrow lettering, and even when sized appropriately, is often challenging to read. In contrast, fonts like Helvetica and Open Sans have wider typefaces and are better suited for study materials. |
| Quinn et al, 2012 | Use large print in document |
| Simonds et al, 2017 | 12 point at least, and consider larger given audience |
| Simonds et al, 2017 | Underline, bold, or boxes (rather than all caps or italics) to give emphasis. |
| Simonds et al, 2017 | Use black Arial or similar font |
| Tait et al, 2013 | Larger font size (14) |
| Tait et al, 2013 | Highlighting using bulleting, bolding and underlining |
| Tait et al, 2005 | Use font size of 14 |
| Tait et al, 2005 | Use boldfacing and underlining for emphasis |
| **Whitespace** | |
| Bonevski et al, 2014 | Wide margins |
| Simply put - CDC | Leave lots of white space. White space is the absence of text or visuals on a page. It keeps a page from being cramped, overwhelming, or amateurish. Many professional graphic designers recommend 10 to 35 percent white space per page for print materials. Leave at least 1⁄2 inch to 1 inch of white space around the margins of the page and between columns. Limit the amount of text and visuals on the page. |
| Cohn et al, 2007 | Use white space |
| Coleman et al, 2021 | Use sufficient spacing between lines (1.2–1.5 is recommended). A soft copy of the PIL should be available so that spacing can be altered based on the needs of the trial participant (e.g., visual impairment) |
| Coleman et al, 2021 | Have a clear contrast in colour between the text and background and include 10–35% white space (space without text). |
| Denzen et al, 2012 | Layout - limit the line length to 30-50 characters and spaces; no more than 5 inches of type running horizontally across a page |
| Denzen et al, 2012 | Layout - leading for body text is approximately 120% of the point size (1-2 points larger) |
| Denzen et al, 2012 | Layout - balance white space with text and graphics |
| Head et al, 2022 | Use white space |
| HRA and Cochrane | Leave 'white space' - avoid large sections of unbroken text or long lists. |
| Mayers et al, 2023 | Adjusting kerning (the space between individual letters) and leading (the space between lines of text) can also enhance readability |
| Mayers et al, 2023 | Appropriate use of whitespace is stressed to ensure study materials are accessible and easy to read |
| Simonds et al, 2017 | Leave a 1-inch margin around the entire document. |
| Simonds et al, 2017 | Layout balances white space with words and graphics |
| Tait et al, 2013 | More white space |
| **Information structure** | |
| Simply put - CDC | Give the most important information first |
| Simply put - CDC | Stick to one idea at a time. Develop one idea fully before moving to the next idea. People are confused when materials skip back and forth between topics. |
| Simply put - CDC | Make the cover attractive to your intended audience |
| Simply put - CDC | Show the main message and audience |
| Simply put - CDC | Place the most important information at the beginning and restate it at the end of the document. |
| Simply put - CDC | Organize ideas in the order that your audience will use them |
| Simply put - CDC | Place key information in a text box. Text boxes make it easier to find the most important information on the page |
| Coleman et al, 2021 | Introduce the purpose of the study early and introduce the study from the participants’ perspective (rather than giving facts that are interesting to the researcher). |
| Coleman et al, 2021 | Check that the key message is first. |
| Coleman et al, 2021 | Consider the use of a summary. |
| Coleman et al, 2021 | Text boxes should be used sparingly and only when appropriate to highlight important pieces of information. They should not encourage the reader to skip through the body of the text. |
| Denzen et al, 2012 | Organisation - include important information near the beginning |
| Jilka et al, 2021 | Ensure order of text makes sense |
| King's fund | Follow the sequence of the patient's own decision-making process |
| King's fund | Group related items of information together |
| King's fund | Offer readers a clear, hierarchical structure with clear signposts for navigation |
| King's fund | Provide a clear, concise overview on the front page |
| King's fund | Structure allows patients to choose a level of detail matching their particular needs and preferences |
| King's fund | Summarise the timetable of clinic visits in a single page 'at a glance' guide |
| Lentz et al, 2016 | CTTI recommends the use of a tiered approach in developing the ICD. The first tier of the document should contain only the basic elements of IC required by federal regulation. Those developing the ICD must critically assess whether information in this tier is truly required. The second tier of the ICD should contain additional information, in chapter format, on a range of study-related issues for each study participant to review as he or she deems necessary. As some participants may elect to bypass this section, information that is critical to the decision-making process should not be introduced for the first time in the second tier. An optional introductory tier consisting of a 1- to 2-page summary of the study may be valuable for complex studies. |
| Mayers et al, 2023 | Organizing study brochure content in such a way that it tells the “story of the study” and includes details that are important to potential participants |
| Quinn et al, 2012 | Give a summary at end of each section |
| Quinn et al, 2012 | Use a question and answer format |
| Simonds et al, 2017 | Break up the text into short straightforward sections |
| Simonds et al, 2020 | Reduce the length and amount of detail by splitting the document into two parts: a general overview followed by a more detailed presentation |
| **Information volume** | |
| Simply put - CDC | Focus on what your audience needs to know and do. Skip details that are only nice to know |
| Simply put - CDC | Limit the number of messages |
| Coleman et al, 2021 | Consider what information the participant would want or need to know. |
| Flory et al, 2024 | Brevity and elimination of irrelevant "boilerplate" information |
| Hughson et al, 2016 | Decrease content |
| Jilka et al, 2021 | Do not make it too long |
| Jilka et al, 2021 | Avoid repetition |
| Lentz et al, 2016 | Limit the amount of information presented |
| Lorell et al, 2015 | Make shorter |
| Tait et al, 2013 | Shortened form |
| **Images** | |
| Adams et al, 2007 | Use of illustrations and other types of graphics amongst populations with little or no formal education greatly aided comprehension |
| Atwere et al, 2018 | Graphical elements are clear and clearly labelled |
| Simply put - CDC | Limit the use of symbols. What is meaningful and natural for one audience may be confusing or misleading to others. Pretest any use of symbols |
| Simply put - CDC | Photographs work best for showing “real life” events, people, and emotions. Photographs tend to be more compelling to audiences. When choosing a photo, be sure any background images will not distract your audience from the image you wish to highlight. |
| Simply put - CDC | Simple illustrations or line drawings may work best in some instances. An illustration or drawing can simplify complexities and highlight key components of an idea. |
| Simply put - CDC | Use simple drawings and avoid unnecessary details. Steer clear of abstract illustrations that could be misinterpreted. Simple drawings are useful for showing desired actions or to address abstract subjects. They can be useful among disparate audiences, especially mixed cultural groups. |
| Simply put - CDC | Cartoons may be good to convey humour or set a more casual tone. Use cartoons with caution; not all audiences understand them or take them seriously. |
| Simply put - CDC | Present one message per visual. When you show several messages in one visual, audiences may miss some or all of the messages. |
| Simply put - CDC | Label visual with captions. Be sure visuals and captions are placed near related text. |
| Simply put - CDC | Use visuals that help emphasize or explain the text. Consider the space available and potential use of the visual. Steer clear of visuals that merely decorate or are too abstract. |
| Simply put - CDC | Show the actions you want your audience to take. Avoid choosing images that show what the audience should not do. |
| Simply put - CDC | Use images and symbols familiar to your audience. |
| Simply put - CDC | Place visuals near the text to which they refer. |
| Simply put - CDC | Use brief captions that include your key message |
| Simply put - CDC | When showing a sequence, number the images. |
| Simply put - CDC | Use cues like arrows and circles to point out key information in your visuals |
| Simply put - CDC | Pictographs are pictures that represent words or ideas |
| Simply put - CDC | Use realistic images for context |
| Simply put - CDC | For a sense of scale, draw small objects larger to show detail. |
| Simply put - CDC | Visuals should have a sharp resolution, true colour and contrast, and good composition. |
| Cohn et al, 2007 | Culturally sensitive graphics |
| Coleman et al, 2021 | Use 1–3 simple images or illustrations when appropriate to support the main message of the PIL, or to explain a difficult concept. Each image should be clear and accompanied by a caption. |
| Coleman et al, 2021 | Put the image/graphic next to the text that it refers to. |
| Coleman et al, 2021 | Have one message per image/graphic. |
| Coleman et al, 2021 | Check that images/graphics are explained and captioned |
| Cunningham-Erves et al, 2022 | Use visuals recognised by the target population |
| Dellson et al, 2011 | Use illustrations |
| Denzen et al, 2012 | Use simple graphics that work with the text, are culturally relevant and reproduce well |
| Eeckhout et al, 2023 | Complement PIL with graphics, diagrams and pictograms |
| Foe et al, 2016 | Use pictographs and illustrations |
| Head et al, 2022 | Use infographic |
| HRA and Cochrane collaboration | In some cases, it might be more appropriate to use other media to support the consent process; for example images, diagrams, audio, video or online materials etc |
| Hughson et al, 2016 | Use animations and graphics to enhance comprehension |
| Jilka et al, 2021 | Consider flow diagrams/pictures |
| Lentz et al, 2016 | Incorporate pictograms |
| Lorell et al, 2015 | Use graphics |
| Mayers et al, 2023 | Photos and images are another critical component to designing culturally appropriate, relevant materials, and should be carefully chosen to resonate with and reflect the diversity of potential recruitment populations -choosing photographs that are relatable and approachable, and provides examples reflecting participant diversity across demographics such as race, ethnicity, age, gender, and relationships. |
| Quinn et al, 2012 | Use pictures and illustrations |
| Simonds et al, 2017 | Use photos, graphics or tables if they will help clarify procedures. Use diagrams as helpful additions to narrative |
| Simonds et al, 2020 | Visual images, such as drawings, photographs, tables, and timelines, would make the document easier to understand |
| Tait et al, 2013 | Use pictures and graphs to display risk and benefits |
| Tait et al, 2005 | Use pictographs |
| **Use of numbers and statistics** | |
| Simply put - CDC | Limit use of statistics and use general words like most, many, half. If you must use statistics, try putting them in parentheses. |
| Simply put - CDC | Mathematical concepts, such as risk, normal, and range, may not have meaning to your audience. If possible, use words such as “chance” or “possibility” instead. |
| Coleman et al, 2021 | Consider the use of images/graphics to explain numbers. |
| Coleman et al, 2021 | Check that readers do not have to perform calculations. For example: Say “Take 3 tablets every morning, afternoon and evening for a week” instead of “Take 21 tablets at equal intervals over a 7-day period” |
| Coleman et al, 2021 | Use specific amounts, rather than words like ‘multiple’ – for example. “As part of this study, you will need to come to the hospital six times to see the study team” |
| Coleman et al, 2021 | For the numbers 0–9, use their words, for 10+ use the digit, unless you are giving an example using a statistic. For example: “1 in 6 people will get a skin rash" |
| Coleman et al, 2021 | Use whole numbers to explain risk or benefits – for example “1 in 6 people will get a skin rash”, rather than “16.67%”. Consider using a visual to explain risks – for example: a group of stick figures with one of them in a different colour |
| Coleman et al, 2021 | Check that numbers are explained. |
| **Co-production** | |
| Bodicoat et al, 2021 | Consult with community members regarding study resources |
| Brockhoven et al, 2023 | Research teams should work with representatives from minoritized backgrounds, engagement practitioners, multicultural agencies and community organisations to develop accessible patient information sheets |
| Simply put - CDC | Get advice from community organizations in the areas you wish to reach |
| Cunningham-Erves et al, 2022 | Use materials inclusive of diverse populations - consult members of the target audience to design materials |
| **Inclusive language** | |
| Corneli et al, 2006 | Use of culturally appropriate analogies to explain medical research |
| Jilka et al, 2021 | Use inclusive phrasing |
| **Plain language** | |
| Addissie et al, 2016 | Fewer sentences per paragraph |
| Addissie et al, 2016 | Fewer words per sentence |
| Atwere et al, 2018 | Abbreviations and acronyms are minimally used and well-defined |
| Atwere et al, 2018 | Language should be non-technical as is feasible |
| Beasant et al, 2023 | Use transparent, clear and precise language |
| Benatar et al, 2012 | Remove legalistic language |
| Benatar et al, 2012 | Simplify the study-specific information |
| Bonevski et al, 2014 | Shorten paragraphs |
| Bonevski et al, 2014 | Use plain language |
| Bonevski et al, 2014 | Use shorter sentences |
| Brockhoven et al, 2023 | Use simple and clear language, avoid the use of scientific jargon and complex terminology |
| Burks et al 2019 | Use a benign metaphor for randomisation |
| Burks et al 2019 | Use plain, active, everyday language and avoid using technical jargon |
| Simply put - CDC | Clearly state the actions you want your audience to take |
| Simply put - CDC | Use concrete nouns |
| Simply put - CDC | Use an active voice |
| Simply put - CDC | Highlight the positive |
| Simply put - CDC | Tell your audience what they will gain from understanding and using the material. Tell your audience how your materials will benefit them. Answer the question, “What’s in it for me?” |
| Simply put - CDC | Use words with one or two syllables when you can |
| Simply put - CDC | Keep most sentences, if possible, between eight to ten words |
| Simply put - CDC | Limit paragraphs to three to five sentences |
| Simply put - CDC | Communicate as if you were talking to a friend. A conversational style has a more natural tone and is easy to understand. |
| Simply put - CDC | Respect and value your audience. Don’t talk down or preach. People are less likely to act on information if they are made to feel bad about their current behaviour or health situation |
| Simply put - CDC | Use a tone that encourages the audience. Emphasize small, practical steps. Offer concrete examples of successful action steps. |
| Simply put - CDC | Limit use of jargon, technical, or scientific language. Define necessary jargon or technical terms first. Then explain them in language your audience will understand. |
| Simply put - CDC | Choose words with a single definition or connotation. People with limited literacy skills may not be able to figure out the meaning from the context. |
| Simply put - CDC | Be consistent with word use. Pick the most familiar words and use them throughout your text. |
| Simply put - CDC | Use analogies familiar to your audience. When making comparisons, use references that your audience will recognize. |
| Simply put - CDC | Avoid unnecessary abbreviations and acronyms. Provide the acronym first and then spell the word (s) out in parentheses when using a familiar abbreviation or acronym. |
| Simply put - CDC | Limit use of quotation marks. Choose other formats to show who is speaking when writing dialogue. |
| Simply put - CDC | Use terms that your audience uses and/or is comfortable with. |
| Simply put - CDC | Test for readability |
| Coleman et al, 2021 | Don’t use long paragraphs. Break up blocks of text using subheadings. |
| Coleman et al, 2021 | Use the active voice, wherever possible. Aim for 80–90% active verbs. For example: “The tablets should be taken twice a day”, rather than “Take the tablets twice a day”. |
| Coleman et al, 2021 | Use every day analogies – for example: “Rub a pea-sized amount of the cream into your skin”. These must be clear and easy to interpret. |
| Coleman et al, 2021 | Ask a colleague not familiar with the disease area to assess readability; Carry out user testing, among a group with the target literacy level; Use a readability assessment tool, such as readability software or the function in Microsoft Word to assess reading age, percentage passive voice and mean sentence length. These assessment tools are useful for guidance but have their limitations; Use a recognized checklist. |
| Coleman et al, 2021 | Context should be provided before giving new information – for example: “If you feel ill, phone the research team”. |
| Coleman et al, 2021 | Minimize the use of technical language or jargon, and where it is necessary, explain it immediately after it is used. |
| Coleman et al, 2021 | Minimize the use of abbreviations or acronyms and where they are necessary, explain them immediately after they are used. |
| Coleman et al, 2021 | Use plain, clear, everyday (but not sloppy) language. |
| Coleman et al, 2021 | Avoid adding information using a subordinate clause (or adding to a sentence with a phrase which can’t stand alone) – for example: Say “Keep your eye drops away from sunlight. Sunlight can damage the drops” instead of “Keep your eye drops away from sunlight, because sunlight can damage the drops”. |
| Coleman et al, 2021 | Minimize the use of long sentences. Aim for an average sentence length of 15–20 words. |
| Cunningham-Erves et al, 2022 | Use preferred terminology of the targeted population |
| Dellson et al, 2011 | Use plain language |
| Dellson et al, 2011 | Avoid and if needed, explain medical terms |
| Dellson et al, 2011 | Use short sentences and simple words |
| Denzen et al, 2012 | Paragraphs should be short; convey one idea per paragraph |
| Denzen et al, 2012 | Ensure verbs are in active voice |
| Denzen et al, 2012 | Reading level should be eight-grade or lower |
| Denzen et al, 2012 | Use words that are familiar to the reader, avoid jargon, acryonms, symbols and abbreviations |
| Denzen et al, 2012 | Ensure words and terminology are consistent throughout the document |
| Denzen et al, 2012 | Ensure sentences are short, simple and direct |
| Eeckhout et al, 2023 | Use shorter PIL with less medical jargon and legal language |
| Foe et al, 2016 | Use the active voice |
| Foe et al, 2016 | Simplify words |
| Head et al, 2022 | Provide a clear explanation using plain language, 6th grade reading level |
| HRA and Cochrane | You should test your Participant Information Sheet with an appropriate group of people (patient groups or other members of the public) |
| HRA and Cochrane | Use conversational style in the active voice |
| HRA and Cochrane | Use short, familiar words and short sentences. |
| HRA and Cochrane | Write in simple, non-technical terms that a lay person will easily understand. |
| HRA and Cochrane | The language used should be no more difficult to read than information leaflets for medicines or tabloid newspapers. |
| HRA and Cochrane | You can use the readability statistics function available in Microsoft Word to calculate a readability score. |
| HRA and Cochrane | When technical terms have to be included you may wish to provide the lay term first followed by the technical term in brackets |
| HRA and Cochrane | Limit sentences to no more than approximately 20 words, when possible. |
| HRA and Cochrane | Don't introduce more than one idea/point in a sentence. |
| HRA and Cochrane | If your next sentence does not directly follow the previous one, start a new paragraph. |
| HRA and Cochrane | Avoid potentially misunderstood words (more obscure or commonly misunderstood) or phrases or words with dual or nuanced meanings (e.g. drugs; diet); especially those likely to cause difficulty to those who have English as a second or third language. |
| HRA and Cochrane | Hard words are technical words, jargon, not commonly used words, or words that are long or with many syllables. |
| HRA and Cochrane | Avoid more than 2 hard words in a sentence unless it is a term that is explained (consider introducing an acronym or shorter term for repeated use). |
| Hughson et al, 2016 | Provide simple, appropriate and systematic explanations of research; simplify language |
| Jefford et al, 2008 | Use familiar words and ideas |
| Jefford et al, 2008 | Check readability |
| Jefford et al, 2008 | Use short words; words of three or fewer syllables should be used when possible |
| Jefford et al, 2008 | Use short sentences |
| Jilka et al, 2021 | Avoid large sections of unbroken text |
| Jilka et al, 2021 | Do not use jargon |
| Jilka et al, 2021 | Avoid potentially misunderstood words |
| Lentz et al, 2016 | ICDs should be evaluated using health literacy/plain language assessments, reading level assessments, and usability testing with patients similar to those who would be eligible for the study. |
| Lentz et al, 2016 | It may also be worthwhile to develop and reach consensus on a standard language library for text that is not specific to the study, and is universally accessible and widely acceptable to IRBs, study sponsors, investigators, and others involved in preparation of the ICD. |
| Lorell et al, 2015 | Use simpler and more understandable language - written at the appropriate grade and health literacy level, and explaining technical terms in clear language. |
| Mayers et al, 2023 | Clear, concise, plain-language, personable, and consistent messaging is encouraged across all study materials. Content should be written at a sixth-grade reading level to reach individuals with varying levels of educational attainment, and should contain common, simplified medical terms, with limited use of acronyms. |
| Quinn et al, 2012 | Use plain language |
| Simonds et al, 2017 | Limit paragraphs to one main idea |
| Simonds et al, 2017 | Whenever possible use active voice |
| Simonds et al, 2017 | Write at an 8th graduate reading level or less |
| Simonds et al, 2017 | Use words familiar to the audience. Write consent form in conversational style, as if you were speaking to the reader |
| Simonds et al, 2017 | Lay language should be used. Avoid technical or professional language used in grant submissions or with peers. Define terms which might not be familiar to the average person the first time they are mentioned. Avoid research and medical jargon whenever possible. If you must use a complicated term,vdefine it in plain language and provide an example, an analogy, or a visual aid. Scientific, technical, and medical terms must be defined or explained in lay terms |
| Simonds et al, 2017 | Use short, simple and direct sentences. Use short sentences. Average sentence length of 15 words or less |
| Tait et al, 2013 | Write at an eight grade reading level |
| Tait et al, 2005 | Avoid dense paragraphs |
| Tait et al, 2005 | Use sentences with a readable sentence structure (microprocessing) |
| **Study purpose and invite** | |
| Simonds et al, 2017 | Start with an introductory sentence describing the primary purpose of the research as stated in the protocol: State what the study is designed to discover or establish. |

**References**

1. Adams V, Miller S, Craig S, et al. Informed consent in cross-cultural perspective: Clinical research in the Tibetan Autonomous Region, PRC. *Culture Medicine and Psychiatry* 2007; 31: 445-472. Article. DOI: 10.1007/s11013-007-9070-2.

2. Addissie A, Abay S, Feleke Y, et al. Cluster randomized trial assessing the effects of rapid ethical assessment on informed consent comprehension in a low-resource setting. *Bmc Medical Ethics* 2016; 17: 12. Article. DOI: 10.1186/s12910-016-0127-z.

3. Atwere P, McIntyre L, Carroll K, et al. Informed Consent Documents Used in Critical Care Trials Often Do Not Implement Recommendations. *Critical Care Medicine* 2018; 46: E111-E117. Article. DOI: 10.1097/ccm.0000000000002815.

4. Beasant L, Realpe A, Douglas S, et al. Autistic adults' views on the design and processes within randomised controlled trials: The APRiCoT study. *Autism* 2023: 13623613231202432. 2023/10/26. DOI: 10.1177/13623613231202432.

5. Benatar JR, Mortimer J, Stretton M and Stewart RAH. A Booklet on Participants' Rights to Improve Consent for Clinical Research: A Randomized Trial. *Plos One* 2012; 7: 7. Article. DOI: 10.1371/journal.pone.0047023.

6. Bodicoat DH, Routen AC, Willis A, et al. Promoting inclusion in clinical trials-a rapid review of the literature and recommendations for action. *Trials* 2021; 22: 880. 2021/12/06. DOI: 10.1186/s13063-021-05849-7.

7. Bonevski B, Randell M, Paul C, et al. Reaching the hard-to-reach: a systematic review of strategies for improving health and medical research with socially disadvantaged groups. *BMC Med Res Methodol* 2014; 14: 42. Article 2014/03/29. DOI: 10.1186/1471-2288-14-42.

8. Brockhoven F, Raphael M, Currier J, et al. REPRESENT recommendations: improving inclusion and trust in cancer early detection research. *British Journal of Cancer* 2023; 129: 1195-1208. Article. DOI: 10.1038/s41416-023-02414-8.

9. Burks AC and Keim-Malpass J. Health literacy and informed consent for clinical trials: a systematic review and implications for nurses. *Nursing-Research and Reviews* 2019; 9: 31-40. Review. DOI: 10.2147/nrr.S207497.

10. Cohn E and Larson E. Improving participant comprehension in the informed consent process. *J Nurs Scholarsh* 2007; 39: 273-280. Article 2007/09/01. DOI: 10.1111/j.1547-5069.2007.00180.x.

11. Coleman E, O'Sullivan L, Crowley R, et al. Preparing accessible and understandable clinical research participant information leaflets and consent forms: a set of guidelines from an expert consensus conference. *Res Involv Engagem* 2021; 7: 31. 2021/05/20. DOI: 10.1186/s40900-021-00265-2.

12. Corneli AL, Bentley ME, Sorenson JR, et al. Using formative research to develop a context-specific approach to informed consent for clinical trials. *Journal of Empirical Research on Human Research Ethics* 2006; 1: 45-60. Article. DOI: 10.1525/jer.2006.1.4.45.

13. Cunningham-Erves J, Kusnoor SV, Villalta-Gil V, et al. Development and pilot implementation of guidelines for culturally tailored research recruitment materials for African Americans and Latinos. *Bmc Medical Research Methodology* 2022; 22: 14. Article. DOI: 10.1186/s12874-022-01724-4.

14. Dellson P, Nilbert M and Carlsson C. Patient representatives' views on patient information in clinical cancer trials. *BMC health services research* 2016; 16: 36. 20160201. DOI: 10.1186/s12913-016-1272-2.

15. Dellson P, Nilbert M, Bendahl PO, et al. Towards optimised information about clinical trials; identification and validation of key issues in collaboration with cancer patient advocates. *Eur J Cancer Care (Engl)* 2011; 20: 445-454. 20100826. DOI: 10.1111/j.1365-2354.2010.01207.x.

16. Denzen EM, Santibáñez MEB, Moore H, et al. Easy-to-Read Informed Consent Forms for Hematopoietic Cell Transplantation Clinical Trials. *Biology of Blood and Marrow Transplantation* 2012; 18: 183-189. Review. DOI: 10.1016/j.bbmt.2011.07.022.

17. Eeckhout D, Aelbrecht K and Van der Straeten C. Informed Consent: Research Staff's Perspectives and Practical Recommendations to Improve Research Staff-Participant Communication. *Journal of Empirical Research on Human Research Ethics* 2023; 18: 3-12. Article. DOI: 10.1177/15562646221146043.

18. Flory J and Emanuel E. Interventions to improve research participants' understanding in informed consent for research: a systematic review. *JAMA : the journal of the American Medical Association* 2004; 292: 1593-1601. DOI: 10.1001/jama.292.13.1593.

19. Foe G and Larson EL. Reading Level and Comprehension of Research Consent Forms: An Integrative Review. *J Empir Res Hum Res Ethics* 2016; 11: 31-46. DOI: 10.1177/1556264616637483.

20. Head KJ, Hartsock JA, Bakas T, et al. Development of Written Materials for Participants in an Alzheimer's Disease and Related Dementias Screening Trial. *J Patient Exp* 2022; 9: 23743735221092573. 20220412. DOI: 10.1177/23743735221092573.

21. Hughson JA, Woodward-Kron R, Parker A, et al. A review of approaches to improve participation of culturally and linguistically diverse populations in clinical trials. *Trials* 2016; 17: 263. 20160526. DOI: 10.1186/s13063-016-1384-3.

22. Jefford M and Moore R. Improvement of informed consent and the quality of consent documents. *Lancet Oncol* 2008; 9: 485-493. DOI: 10.1016/S1470-2045(08)70128-1.

23. Jilka S, Hudson G, Jansli S, et al. How to make study documents clear and relevant: the impact of patient involvment. *BJPsych Open* 2021; 7: 1-8.

24. Kass NE, Taylor HA, Ali J, et al. A pilot study of simple interventions to improve informed consent in clinical research: feasibility, approach, and results. *Clin Trials* 2015; 12: 54-66. 20141204. DOI: 10.1177/1740774514560831.

25. Lentz J, Kennett M, Perlmutter J and Forrest A. Paving the way to a more effective informed consent process: Recommendations from the Clinical Trials Transformation Initiative. *Contemp Clin Trials* 2016; 49: 65-69. 20160617. DOI: 10.1016/j.cct.2016.06.005.

26. Lorell BH, Mikita JS, Anderson A, et al. Informed consent in clinical research: Consensus recommendations for reform identified by an expert interview panel. *Clin Trials* 2015; 12: 692-695. 20150715. DOI: 10.1177/1740774515594362.

27. Mayers SA, Cook SK, Rantala C, et al. The RIC Recruitment & Retention Materials Toolkit - a resource for developing community-informed study materials. *J Clin Transl Sci* 2023; 7: e182. 20230807. DOI: 10.1017/cts.2023.607.

28. Quinn SC, Garza MA, Butler J, et al. Improving informed consent with minority participants: results from researcher and community surveys. *J Empir Res Hum Res Ethics* 2012; 7: 44-55. DOI: 10.1525/jer.2012.7.5.44.

29. Simonds VW, Garroutte EM and Buchwald D. Health Literacy and Informed Consent Materials: Designed for Documentation, Not Comprehension of Health Research. *J Health Commun* 2017; 22: 682-691. 20170731. DOI: 10.1080/10810730.2017.1341565.

30. Simonds VW and Buchwald D. Too Dense and Too Detailed: Evaluation of the Health Literacy Attributes of an Informed Consent Document. *J Racial Ethn Health Disparities* 2020; 7: 327-335. 20191210. DOI: 10.1007/s40615-019-00661-1.

31. Spellecy R, Tarima S, Denzen E, et al. Easy-to-Read Informed Consent Form for Hematopoietic Cell Transplantation Clinical Trials: Results from the Blood and Marrow Transplant Clinical Trials Network 1205 Study. *Biol Blood Marrow Transplant* 2018; 24: 2145-2151. 20180418. DOI: 10.1016/j.bbmt.2018.04.014.

32. Tait AR, Voepel-Lewis T, Malviya S and Philipson SJ. Improving the readability and processability of a pediatric informed consent document: effects on parents' understanding. *Arch Pediatr Adolesc Med* 2005; 159: 347-352. DOI: 10.1001/archpedi.159.4.347.

33. Tait AR, Voepel-Lewis T, Nair VN, et al. Informing the uninformed: optimizing the consent message using a fractional factorial design. *JAMA Pediatr* 2013; 167: 640-646. DOI: 10.1001/jamapediatrics.2013.1385.
